# Correction to “Cytocompatible Triblock Copolymers with Controlled Microstructure Enabling Orthogonally Functionalized Bio-polymer Conjugates”

**DOI:** 10.1021/acs.macromol.4c00692

**Published:** 2024-04-19

**Authors:** Kerstin Halama, Molly Tzu-Yu Lin, Andreas Schaffer, Marvin Foith, Friederike Adams, Bernhard Rieger

**Affiliations:** † WACKER-Chair of Macromolecular Chemistry, Catalysis Research Center, Department of Chemistry, Technical University of Munich, Lichtenbergstr. 4, 85748 Garching, Germany; ‡ University Eye Hospital Tübingen, Elfriede-Aulhorn-Strasse 7, 72076 Tübingen, Germany; § Chair of Macromolecular Materials and Fiber Chemistry, Institute of Polymer Chemistry, University of Stuttgart, Pfaffenwaldring 55, 70569 Stuttgart, Germany

In the manuscript and the Supporting Information, the affiliation of Molly Tzu-Yu Lin and Friederike Adams needs to be amended. Their correct affiliation is as follows: University Eye Hospital Tübingen, Elfriede-Aulhorn-Strasse 7, 72076 Tübingen, Germany. This change is reflected in the authorship of this Correction.

The Supporting Information has been expanded to include the following sentence: “The human Müller cell line Moorfields/Institute of Ophthalmology-Müller 1 was obtained from the UCL Institute of Ophthalmology, London, UK.”

This addition necessitates a modification to the text, which now reads: “Cell Viability Assay was used for evaluating the biocompatibility of polymers with spontaneously immortalized human Müller cell line (MIO-M1). The human Müller cell line Moorfields/Institute of Ophthalmology-Müller 1 was obtained from the UCL Institute of Ophthalmology, London, UK. The polymers were prepared at a stock concentration of 1.5 mg/mL in distilled water and vortexed until dissolved before use. MIO-M1 (P41) was seeded in a transparent 96-well plate at a density of 10,000 cells in prewarmed high glucose (4.5 g/L) DMEM medium (Gibco; ThermoFisher Scientific, Taufkirchen, Germany) supplemented with 10% fetal bovine serum and 1% penicillin/streptomycin (ThermoFisher Scientific, Karlsruhe, Germany).”

Additionally, reference 8 has been added:

(8) LimbG. A.; SaltT. E.; MunroP. M.; MossS. E.; KhawP. T.Investigative Ophthalmology & Visual Science2002, 43, 864–869.11867609

## Supporting Information Available

Synthetic procedures of the synthesis, polymerization, and follow-up functionalization; detailed characterization data (^1^H-, ^13^C-, ^31^P-, and DOSY-NMR spectra, fluorescence spectra, UV–vis spectra, DLS measurements, elemental analysis, SEC-MALS traces, as well as cell viability assays) (PDF)

