# Supplementary material for: Correction to “Cytocompatible Triblock Copolymers with Controlled Microstructure Enabling Orthogonally Functionalized Bio-polymer Conjugates”
Source: Macromolecules. 2024 Apr 19;57(9):4635. doi: 10.1021/acs.macromol.4c00692 (PMC11099997; doi:10.1021/acs.macromol.4c00692)
Supplement: Supplementary file 1 — ma4c00692_si_001.pdf [file ma4c00692_si_001.pdf]

Cytocompatible Triblock Copolymers with Controlled Microstructure enabling Orthogonally Functionalized Bio-Polymer-Conjugates

Kerstin Halama[a], Molly Tzu-Yu Lin[b], Andreas Schaffer[a], Marvin Foith[a], Friederike Adams[b,c], Bernhard Rieger[a]*

[a] WACKER-Chair of Macromolecular Chemistry, Catalysis Research Center, Department of Chemistry, Technical University of Munich, Lichtenbergstr. 4, 85748 Garching (Germany)

[b] University Eye Hospital Tübingen, Elfriede-Aulhorn-Strasse 7, 72076 Tübingen, Germany

[c] Chair of Macromolecular Materials and Fiber Chemistry, Institute of Polymer Chemistry, University of Stuttgart, Pfaffenwaldring 55, 70569 Stuttgart, Germany

TABLE OF CONTENTS

1. General Experimental 3
2. Synthesis procedures 6
3. Polymerization procedure 9
4. Post-polymerization functionalization 13
5. References 34

1. GENERAL EXPERIMENTAL

All reactions and polymerizations with moisture and air-sensitive reactants were carried out in an M*Braun* LabMaster120 glovebox filled with argon 4.6 from *Westfalen* or using standard *Schlenk* techniques. All glassware was heat-dried before use. All chemicals were purchased from *Sigma-Aldrich*, ABCR, or TCI Europe and used without further purification unless otherwise stated. Dichloromethane, tetrahydrofuran, toluene, and pentane were dried using an M*Braun* SPS-800 solvent purification system and stored over a 3 Å molecular sieve. Diethyl vinyl phosphonate and diallyl vinyl phosphonate were dried over CaH_2 for several days and distilled before use. $Y(CH_2TMS)_3(thf)$[1], the precursor catalyst $Cp_2Y(CH_2TMS)(thf)$[2], diallyl vinyl phosphonate[3], dichloro vinyl phosphonate[4], (3-(trimethylsilyl)propargyl alcohol[5], thiocholesterol[6] and azido-functionalized folate[7] were prepared according to literature procedures.

Cell Viability Assay was used for evaluating the biocompatibility of polymers with spontaneously immortalized human *Müller* cell line (MIO-M1). The human Müller cell line Moorfields/Institute of Ophthalmology-Müller 1 was obtained from the UCL Institute of Ophthalmology, London, UK.[8] The polymers were prepared at a stock concentration of 1.5 mg/mL in distilled water and vortex until dissolved before use. MIO-M1 (P41) was seeded in a transparent 96-well plate at the density of 10,000 cells in pre-warmed high glucose (4.5 g/L) DMEM medium (Gibco; Thermo*Fisher* Scientific, Taufkirchen, Germany) supplemented with 10% fetal bovine serum and 1% penicillin/streptomycin (Thermo*Fisher* Scientific, Karlsruhe, Germany). After 24 hours, the medium was aspirated, and each well was treated with 100 µL of different concentrations of polymers (5, 15, 25, 50, 100, 150, 200, 250, and 500 µg/mL) prepared in the medium. Following 24 hours of treatment at 37°C and 5% CO_2, an MTS assay was carried out to evaluate the metabolic activity of the treated cells by adding 20 µL of the CellTiter 96 ® Aqueous One Solution Reagent

(Promega Corporation, Madison, WI, USA) directly to each well and incubated at 37°C for 90 minutes. Subsequently, the absorbance was measured at 490 nm with a reference wavelength set at 690 nm for background correction using a a Tecan Reader (NanoQuant infinite M200). All data is shown as mean ± SD from five replicates after normalizing to the untreated control wells representing 100% cell viability.

Dynamic light scattering (DLS) was performed at a Zetasizer Nano ZS (*Malvern*). The diameter was averaged over three independent values consisting each of 10 measurements. The samples were dissolved in water at a concentration of 2.5 mg/mL.

Elemental analysis (EA) was performed by the Laboratory of Microanalytics at the Institute of Inorganic Chemistry at the Technical University of Munich, Department of Chemistry, Catalysis Research Center.

Electrospray Ionization Mass Spectrometry (ESI-MS) was measured using a Thermo *Fisher* Scientific Exactive Plus Orbitrap in a positive mode in HPLC methanol straight from the reaction mixture without quenching.

Fluorescence spectroscopy was performed on a Jasco (Jasco FP-8300, Spectra Manager software 2.13). The concentration of polymers in methanol was determined to be 1.0 mg/mL. Corresponding substrates were prepared in 0.5 mM solutions. Fluorescence spectroscopy was performed on a Jasco (Jasco FP-8300, Spectra Manager software 2.13).

Lyophilization was performed on a VaCO 5-II-D at a pressure of 1 mbar and -90 °C condenser temperature from either 1,4-dioxane or water.

Nuclear magnetic resonance (NMR) spectra were recorded on a Bruker AV-400HD, AV-500HD, or AV-II-500 spectrometer at 400 or 500 MHz (^{1}H), 125 MHz (^{13}C), 203 MHz (^{31}P) and 99 MHz (^{29}Si). NMR spectroscopical shifts δ were reported in ppm relative to the deuterated solvent's residual proton or carbon signal. Deuterated solvents ($CDCl_3$, benzene-d_6, MeOD, DMSO-d_6) were purchased from Sigma-Aldrich or Deutero and dried over 3 Å molecular sieves before use. DOSY-NMR measurements were performed for the characterization of polymer conjugates.

Size-exclusion chromatography multi-angle light scattering (SEC-MALS) was used to determine molecular weights and polydispersity of the polymers (c = 2.5 mg/mL) with a Wyatt Dawn Heleos II MALS light scattering unit and a Wyatt Optilab rEX 536 RI unit in THF: H_2O = 1:1 (with 9 g/L *tetra*-*n*-butyl-ammonium bromide and 272 mg/L 2,6-di-*tert*-butyl-4-methylphenol added) as eluent at 40 °C on two Agilent PolarGel-M columns; for absolute molecular weight (triple detection) determination of the polyvinyl phosphonates the refractive index increment dn/dc = 0.0922 mL/g[9] was used.

UV/vis spectroscopy (UV/vis) was measured on a Varian Cary 50 Scan UV visible spectrophotometer. Spectra were measured from 200 nm to 800 nm at 25 °C. The samples were dissolved in methanol and analyzed in a QS 10×10 mm quartz glass cuvette from *Hellma* GmbH & Co. KG. A baseline correction with the pure solvent was performed before sample measurements.

2. SYNTHESIS PROCEDURES

DI (3-(TRIMETHYLSILYL)PROP-2-YN-1-YL) VINYL PHOSPHONATE (DPRTMSVP)

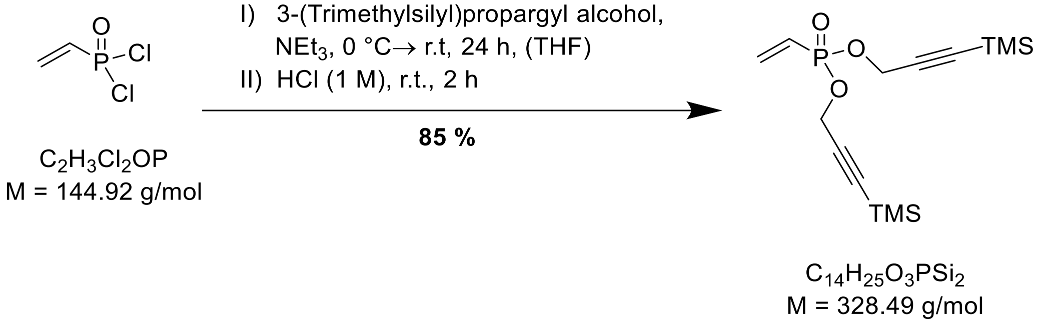

5.15 mL (7.25 g, 50.0 mmol, 1.0 eq.) vinyl phosphonic dichloride and 16.3 mL (14.1 g, 110 mmol, 2.2 eq.) were dissolved in 250 mL of THF in a *Schlenk* flask. Under vigorous stirring, 27.8 mL (20.2 g, 200 mmol, 4.0 eq.) triethyl amine was added at 0 °C. After stirring the solution overnight at room temperature, the reaction course was monitored via ^{31}P NMR spectroscopy. With complete conversion, the reaction is then quenched with 50 mL of water. The solution was filtered and washed with water (2 × 100 mL). The aqueous phase was extracted with ethyl acetate (2 × 200 mL). The combined organic phases were dried over sodium sulfate before the solvent was removed in vacuo. Purification via sublimation with dry iced cooling (75.0 °C, p= 5.0×10^{-6} mbar) yielded 14.0 g (42.5 mmol, 85%) di (3-(trimethylsilyl) prop-2-yn-1-yl)vinyl phosphonate as light yellow oil.

^{1}H-NMR (500 MHz, $CDCl_3$, 300K): δ (ppm) = 6.37 – 5.90 (m, 3H, CH_{Vinyl}), 4.36 (q, 4H, CH_2), 0.12 (s, 18H, $Si(CH_3)_3$).

^{13}C-NMR (126 MHz, $CDCl_3$, 300K): δ (ppm) = 136.4 (s, C_{Vinyl}), 125.1 (d, J_{PC}= 185.6 Hz, PC_{Vinyl}), 99.2 (s, C), 92.9 (s, Si-C), 54.4 (s, CH_2), -0.4 (s, CH_3).

^{31}P-NMR (203 MHz, $CDCl_3$, 300K): δ (ppm) = 19.5.

^{29}Si-NMR (99 MHz, $CDCl_3$, 300K): δ (ppm) = -17.1.

ESI-MS: calculated: 329.11 $[M–H]^+$, found: 329.11 $[M–H]^+$.

EA: calculated: C 51.19%, H 7.67%, P 9.43%.

found: C 51.20%, H 7.74%, P 9.42%.

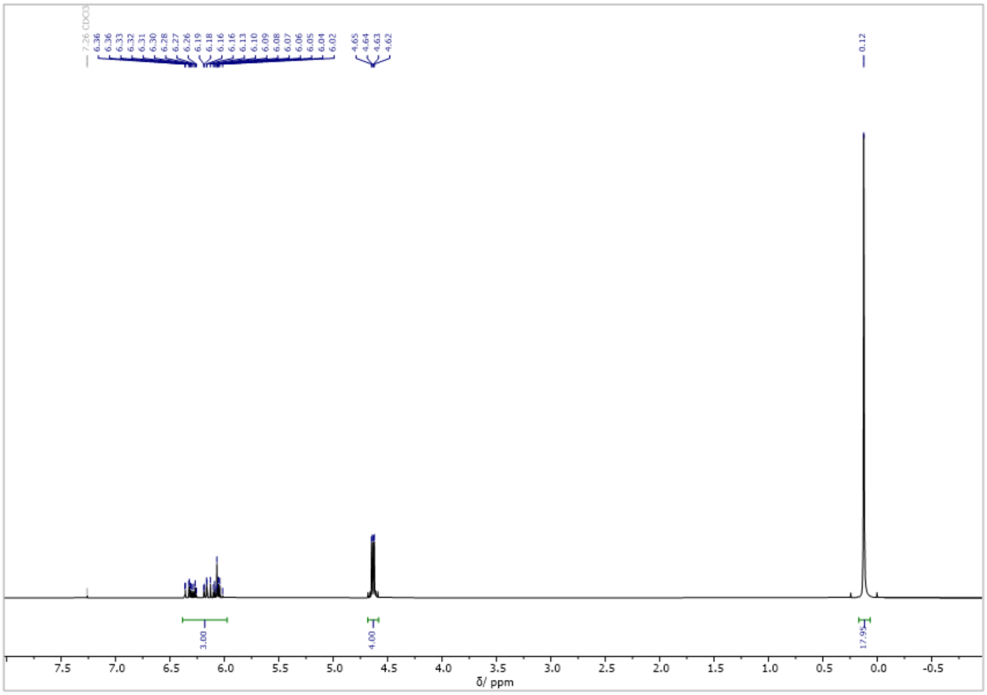

Figure S1: ^{1}H-NMR (500 MHz, $CDCl_3$, 300 K) of di (3-(trimethylsilyl)prop-2-yn-1-yl) vinyl phosphonate (DPrTMSVP).

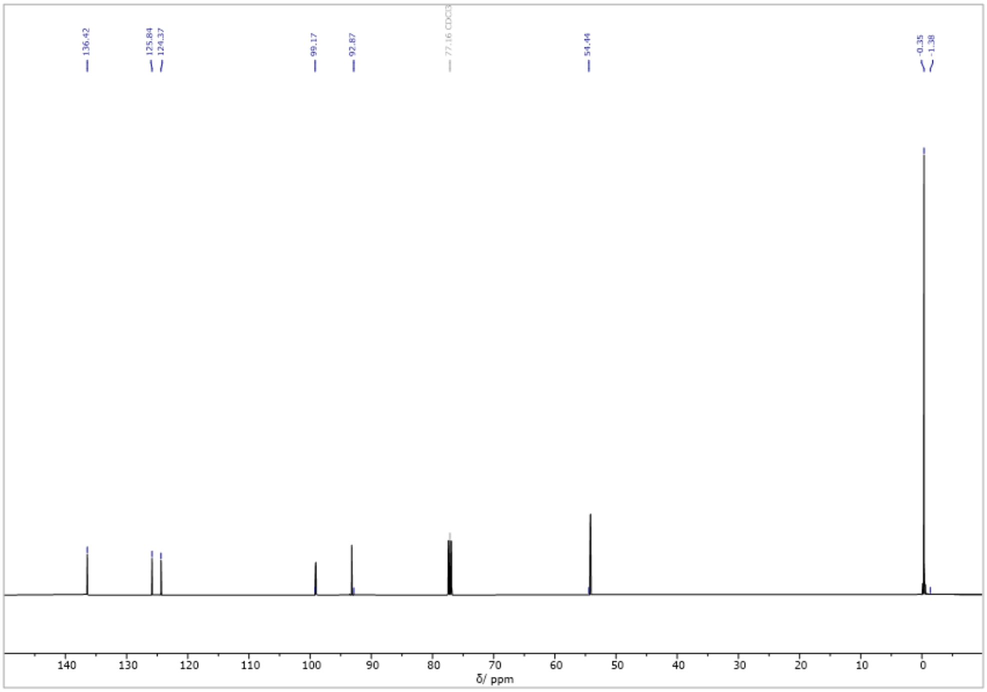

Figure S2: ^{13}C-NMR (126 MHz, $CDCl_3$, 300 K) of di (3-(trimethylsilyl)prop-2-yn-1-yl) vinyl phosphonate (DPrTMSVP).

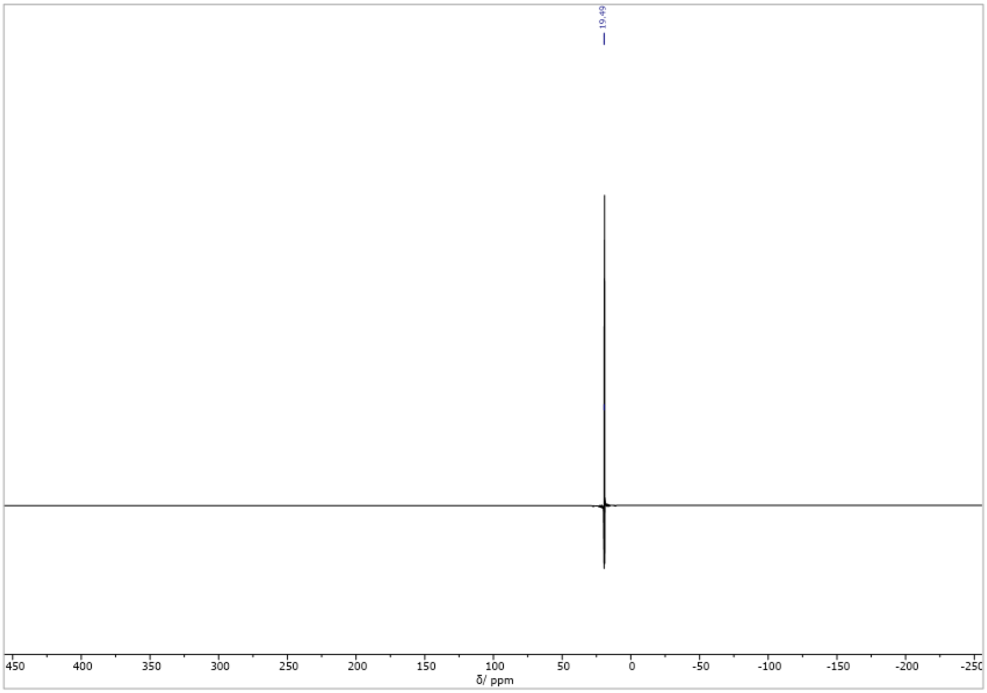

Figure S3: ^{31}P-NMR (203 MHz, $CDCl_3$, 300 K) of di (3-(trimethylsilyl)prop-2-yn-1-yl) vinyl phosphonate (DPrTMSVP).

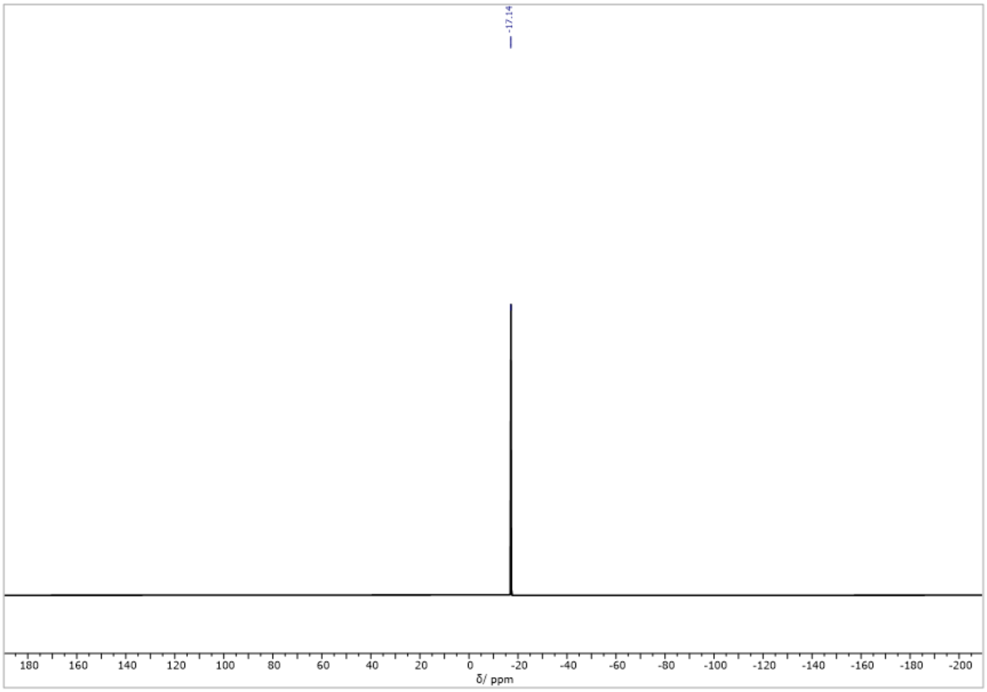

Figure S4: ^{29}Si-NMR (99 MHz, $CDCl_3$, 300 K) of di (3-(trimethylsilyl)prop-2-yn-1-yl) vinyl phosphonate (DPrTMSVP).

3. POLYMERIZATION PROCEDURE

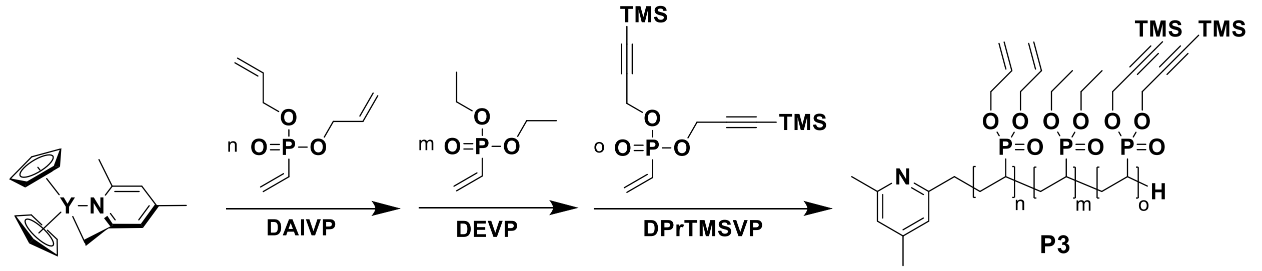

For the C–H bond activation, 75.7 mg (200 µmol, 1.0 eq.) $Cp_2Y(CH_2TMS)(thf)$ was dissolved in 5.0 mL absolute toluene, and 24.3 mg (200 µmol, 1.0 eq.) *sym*-collidin was added to the solution. The *in-situ* activation of the catalyst is performed at room temperature for two hours. For the polymerization, firstly, 188 mg (1.00 mmol, 5.0 eq.) diallyl vinyl phosphonate was added, and an aliquot of PDAlVP **P1** (0.50 mL) was withdrawn after one minute. Secondly, 10.5 mL absolute toluene was refilled after three minutes, and 2.30 g (14.0 mmol, 80 eq.) diethyl vinyl phosphonate was added. 1.00 mL of the reaction solution is used for an aliquot of P(DAlVP-*co*-DEVP) **P2**. For the final polymerization step, 294 mg (825 µmol, 5.0 eq) di (3-(trimethylsilyl)prop-2-yn-1-yl) vinyl phosphonate was used. The polymerization was performed at room temperature overnight before an aliquot of P(DAlVP-DEVP-DPrTMSVP) **P3** is withdrawn, and the reaction was quenched by adding wet methanol. The polymer participated from pentane, subject to centrifugation, and the supernatant was decanted. The residual polymer is dissolved in 1,4-dioxane before being freeze-dried. The purified polymer is analyzed using $^{1}H/^{31}P$/DOSY-NMR and SEC-MALS.

Polyvinyl phosphonate P3

^{1}H-NMR (400 MHz, $CDCl_3$, 300K): δ (ppm) = 6.88 – 6.60 (m, 2H, $H_{ar,sym\text{-Collidin}}$), 5.87 (s, 14H, $\text{-OCH}_2\text{C}H\text{CH}_2$), 5.33 – 5.04 (m, 28H, $\text{-OCH}_2\text{CHC}H_2$), 4.79 (s, 8H, $\text{-C}H_2\text{C≡CSi(CH}_3)_3)$)), 4.49 (s, 28H, $\text{-OC}H_2\text{CHCH}_2$), 4.08 (s, 404H, $\text{-OC}H_2\text{CH}_3$), 2.87 – 0.96 (m, 936H, backbone, $\text{OC}H_2\text{CH}_3$), 0.13 (s, 36H, $\text{-CH}_2\text{C≡CSi(C}H_3)_3$).

^{31}P-NMR (203 MHz, $CDCl_3$, 300K): δ (ppm) = 33.2.

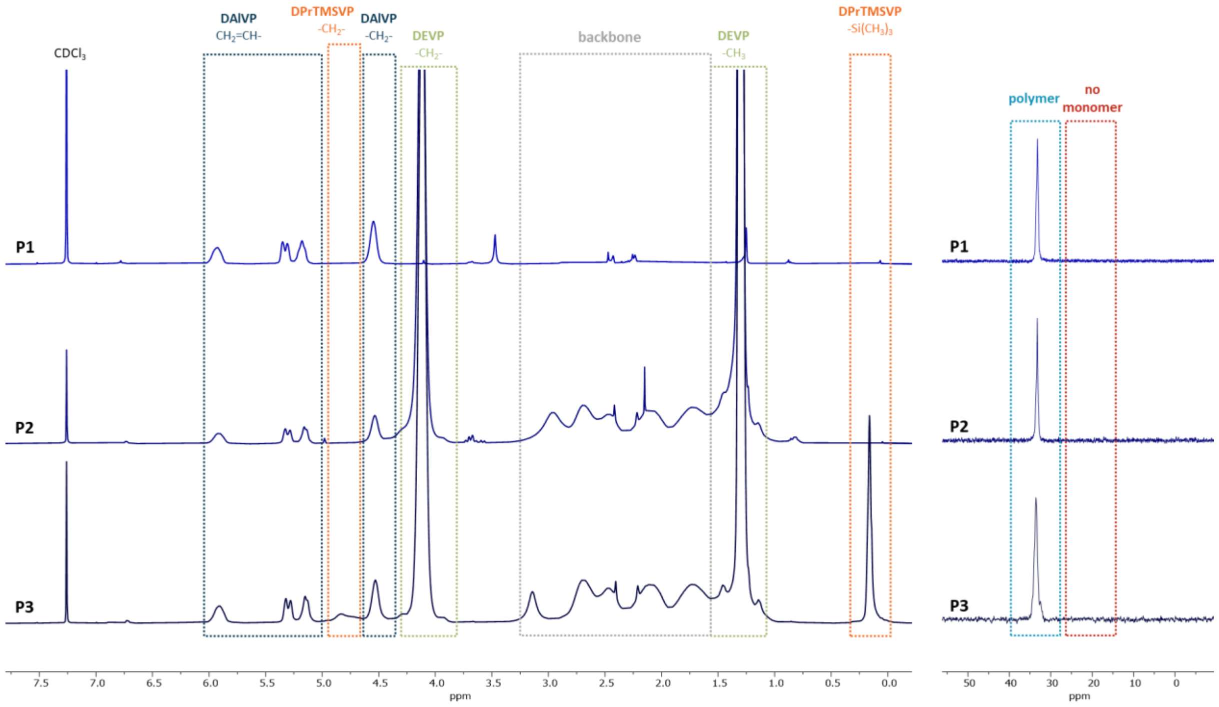

Figure S5: ^{1}H (400 MHz, $CDCl_3$, 300K) (right) and ^{31}P (203 MHz, $CDCl_3$, 300K) (left) of the polyvinyl phosphonates PDAlVP **P1**, P(DAlVP-*co*-DEVP) **P2** and P(DAlVP-DEVP-DPrTMSVP) **P3**.

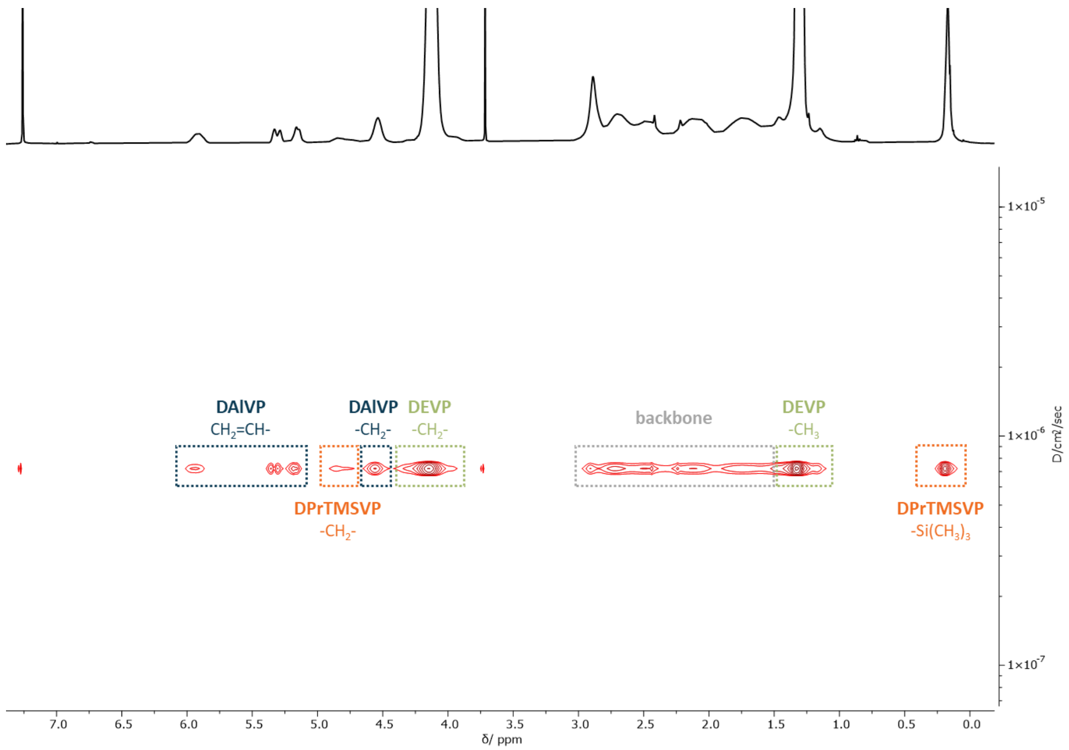

Figure S6: DOSY-NMR of and P(DAlVP-DEVP-DPrTMSVP) **P3.**

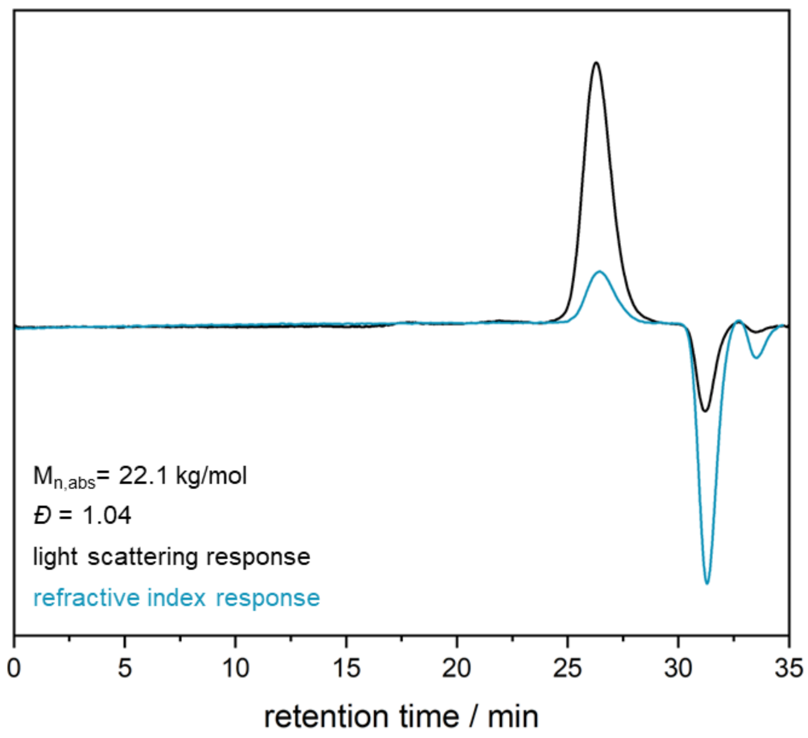

Figure S7: SEC-MALS of P(DAlVP-DEVP) **P2**.

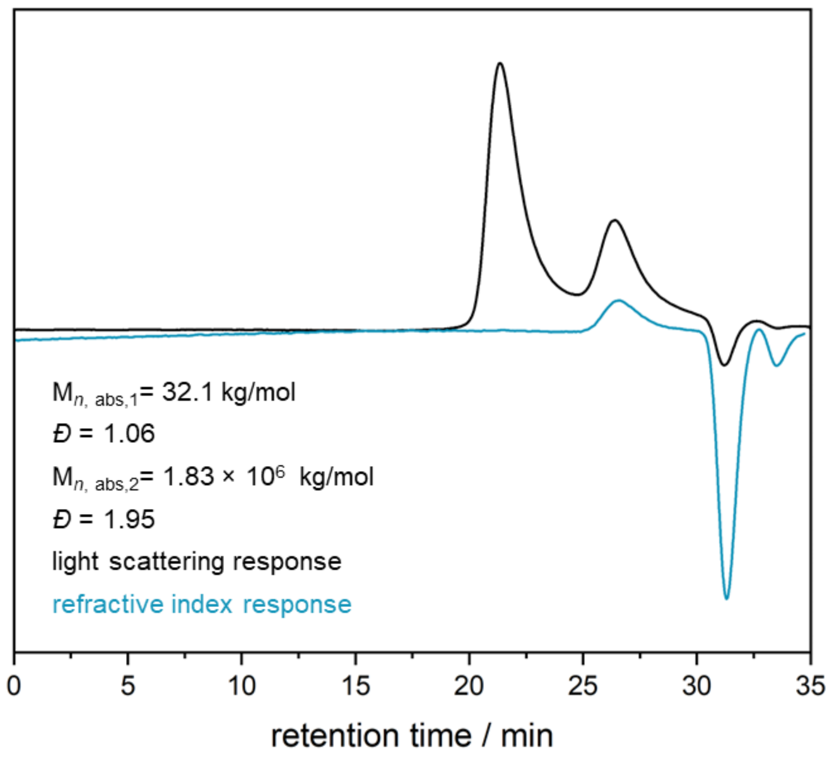

Figure S8: SEC-MALS of P(DAlVP-DEVP-DPrTMSVP) **P3**.

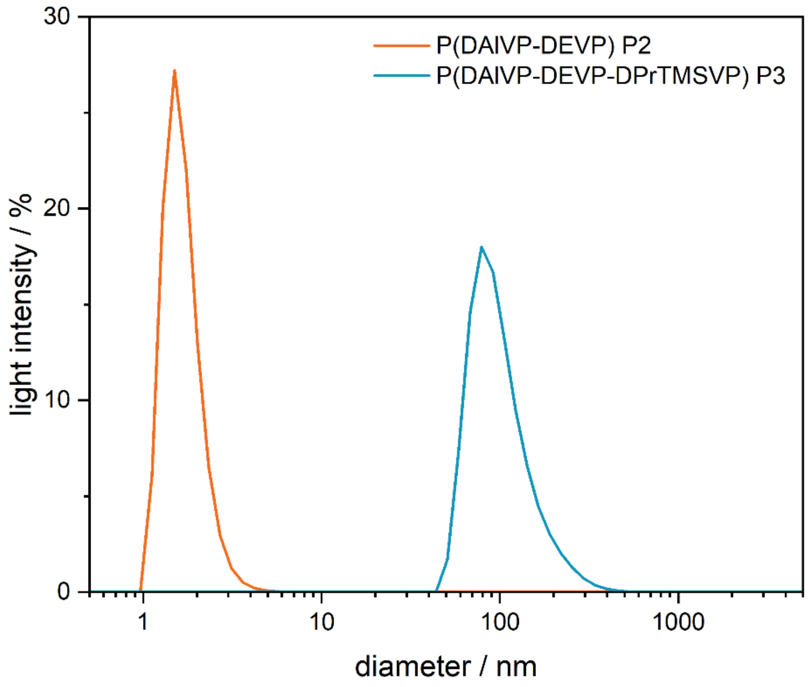

Figure S9: DLS spectra of **P2** (d = 26.5 ± 1.90 nm, PDI = 0.835) and **P3** (d = 128 ± 1.20 nm, PDI = 0.361).

4. POST-POLYMERISATION FUNKTIONALISATION

DEPRODECTION OF PROPARGYL GROUPS (DPRTMSVP)

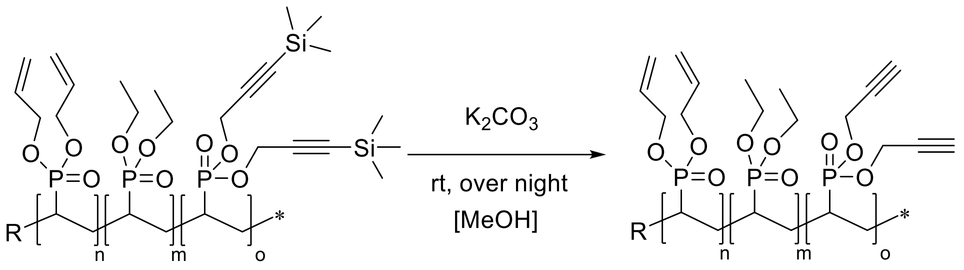

P3 **P4**

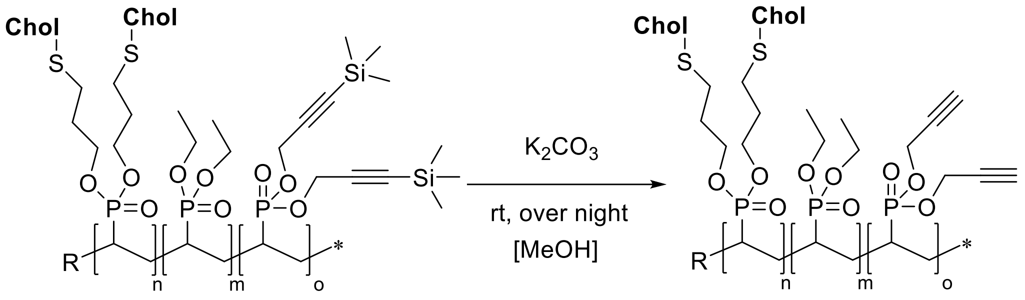

P8 **P9**

For deprotection of the propargyl groups, the polyvinyl phosphonates were dissolved in methanol (5.00 mL solvent per 100 mg polymer), and potassium carbonate (5.0 eq. per propargyl group) was added. The reaction solution was stirred overnight at room temperature before the volatiles were removed under reduced pressure. The residue was purified by dialysis against water and freeze-dried to yield the deprotected polymer.

Deprotection of P3 = P4

^{1}H-NMR (400 MHz, $CDCl_3$, 300K): δ (ppm) = 6.91 – 6.68 (m, $H_{ar,sym\text{-}Collidin}$), 5.92 (s, -$OCH_2C\mathit{H}CH_2$), 5.43 – 5.04 (m, -$OCH_2CHC\mathit{H}_2$), 4.55 (s, -$OC\mathit{H}_2CHCH_2$), 4.13 (s, -$OC\mathit{H}_2CH_3$), 2.87 – 0.96 (m, backbone, $OC\mathit{H}_2CH_3$,-$OCH_2C{\equiv}C\mathit{H}$).

^{31}P-NMR (203 MHz, $CDCl_3$, 300K): δ (ppm) = 33.2.

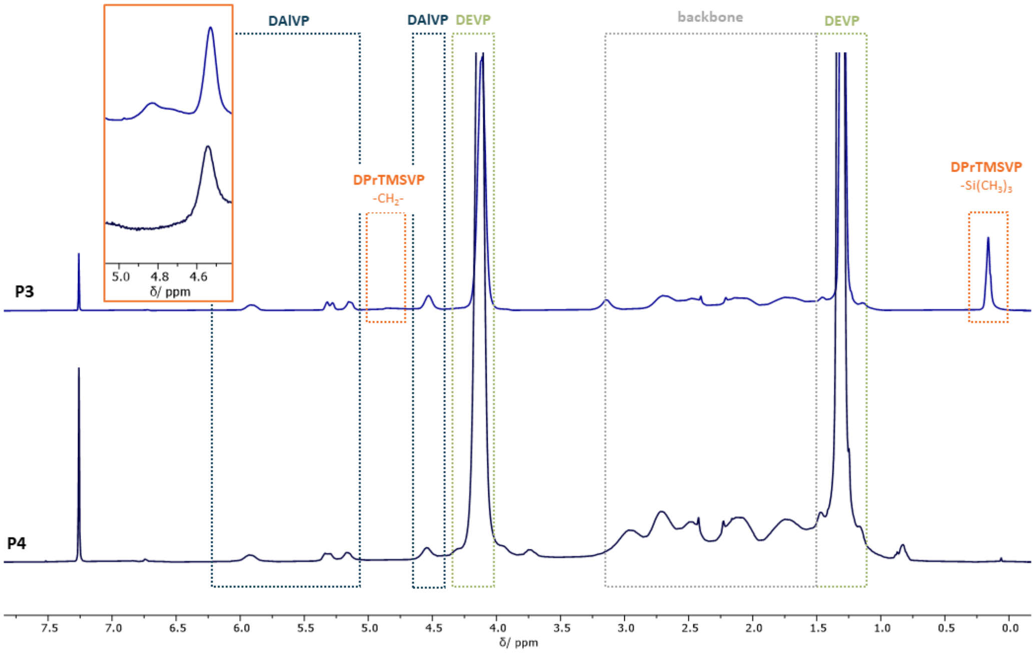

Figure S 10: ^{1}H-NMR (400 MHz, $CDCl_3$, 300K) of the polyvinyl phosphonates P(DAlVP-DEVP-DPrTMSVP) **P3** and P(DAlVP-DEVP-DPrVP) **P4.**

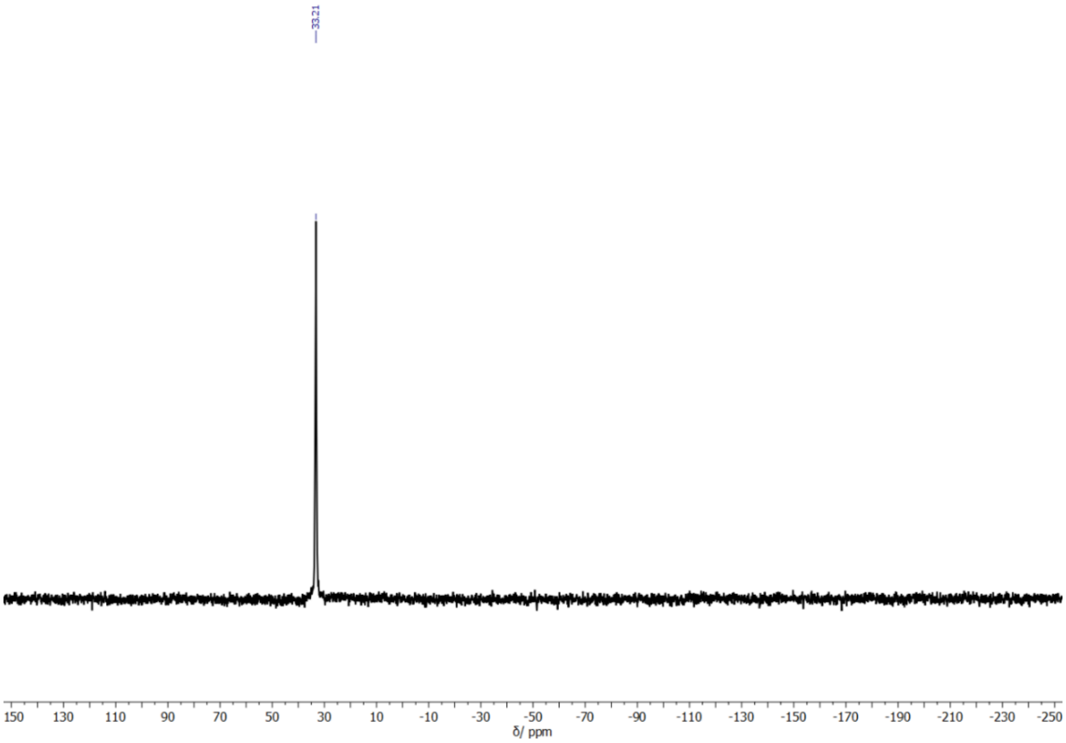

Figure S 11: ^{31}P-NMR (203 MHz, $CDCl_3$, 300K) of P(DAlVP-DEVP-DPrVP) **P4.**

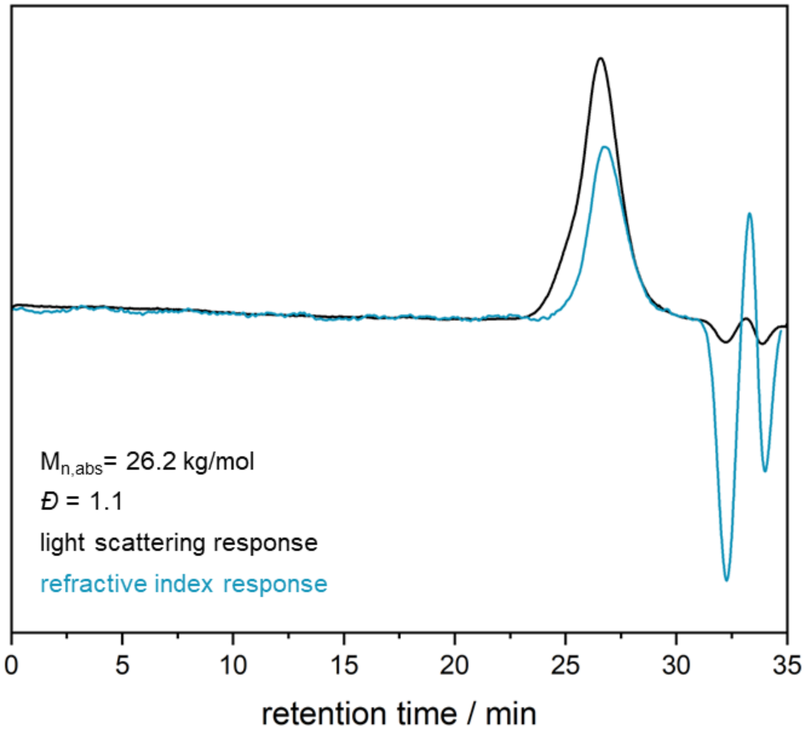

Figure S13: SEC-MALS of P(DAlVP-DEVP-DPrVP) **P4**.

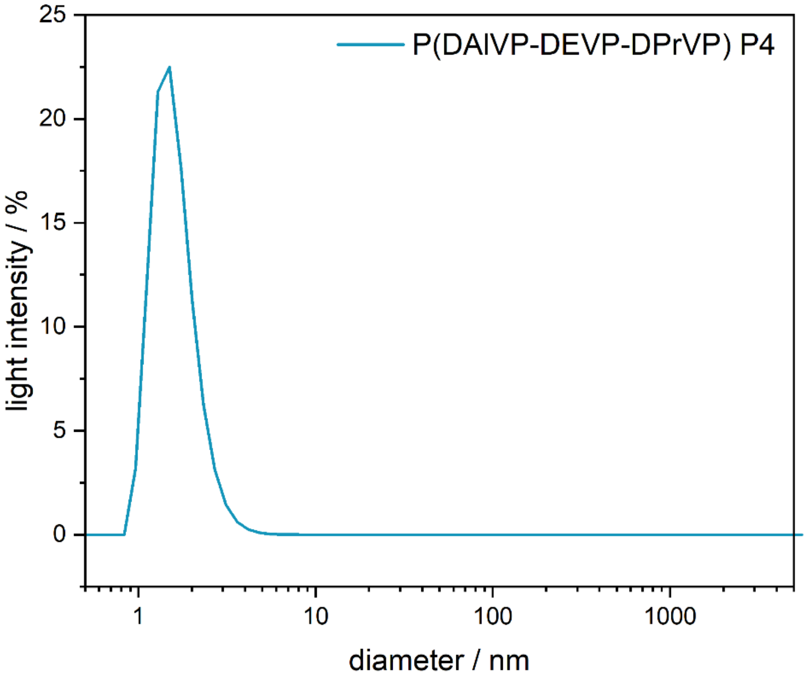

Figure S14: DLS spectra of **P4** (d = 8.10 ± 0.20 nm, PDI = 0.334).

.

Deprotection of P8 = P9

^{1}H-NMR (400 MHz, $CDCl_3$, 300K): δ (ppm) = 6.89 – 6.70 (m, $H_{ar,sym\text{-}Collidin}$), 5.94 (s, -$OCH_2CHCH_2$), 5.54 – 5.13 (m, -$OCH_2CHCH_2$), 4.54 (s, -$OCH_2CHCH_2$), 4.13 (s, -$OCH_2CH_3$), 3.72 (s, -$CH_2C{\equiv}CH$), 2.79 – 0.98 (m, backbone, OCH_2CH_3, $H_{cholesterol}$), 0.99 – 0.81 (m, -$CH_{3,\ cholesterol}$).

^{31}P-NMR (400 MHz, $CDCl_3$, 300K): δ (ppm) = 33.2.

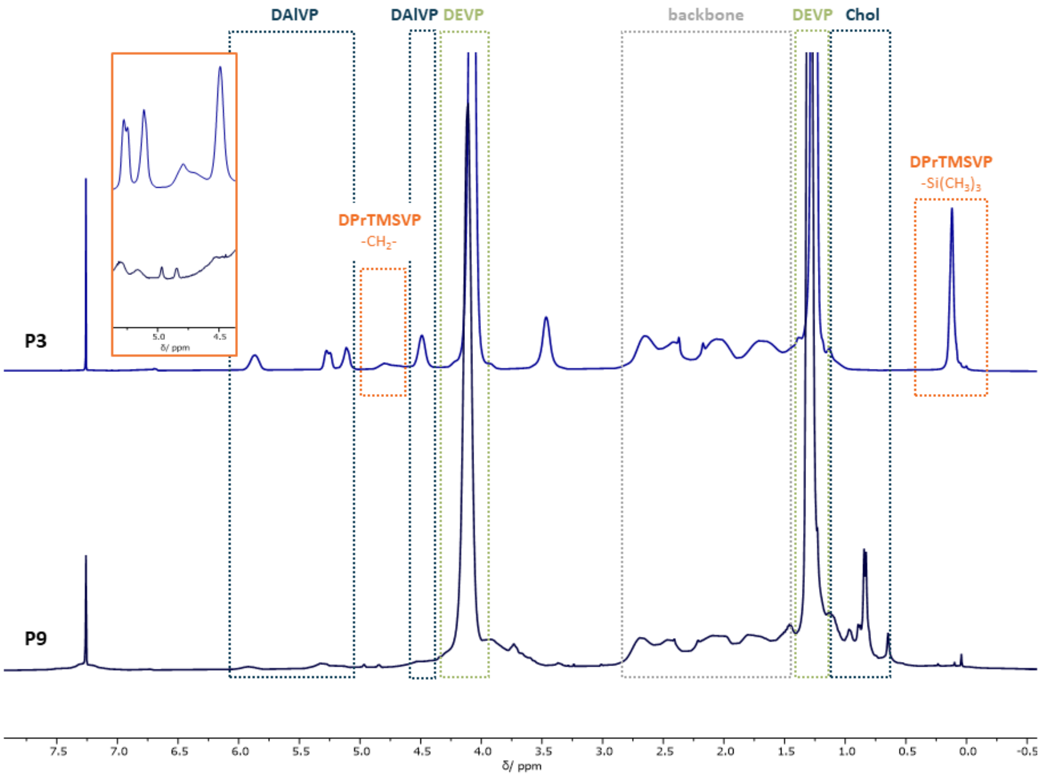

Figure S15: ^{1}H-NMR (400 MHz, $CDCl_3$, 300K) of the polyvinyl phosphonates **P3** and **P9.**

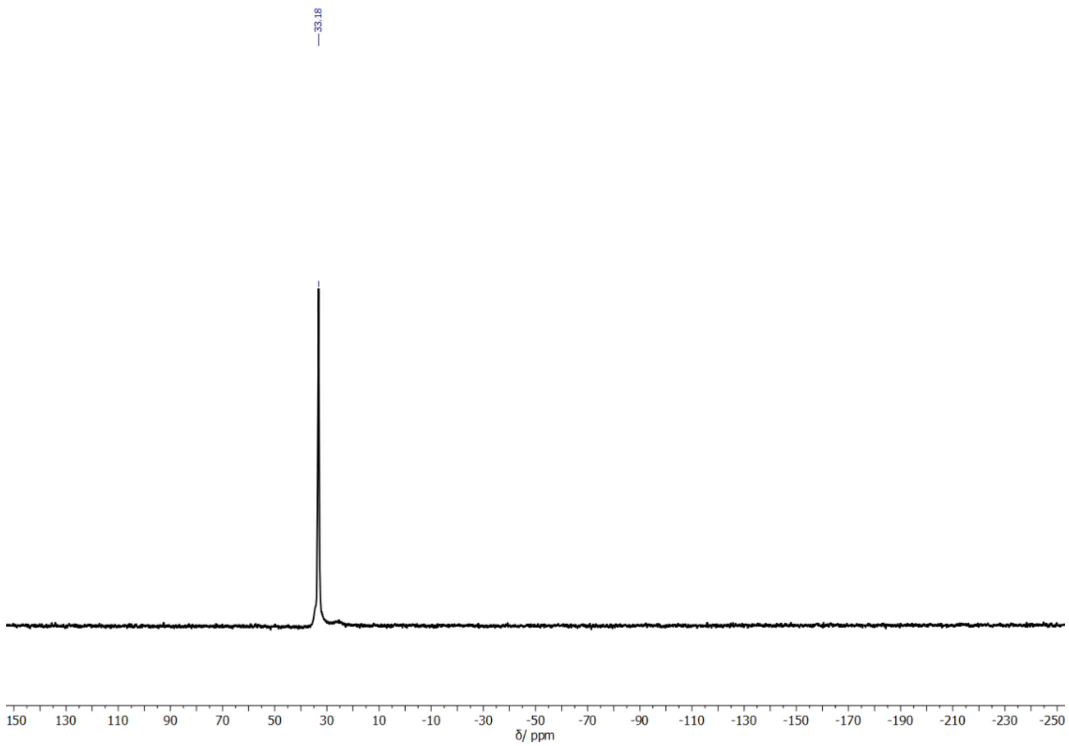

Figure S 16: ^{31}P-NMR (203 MHz, $CDCl_3$, 300K) of P(DAlVP-DEVP-DPrVP) **P9.**

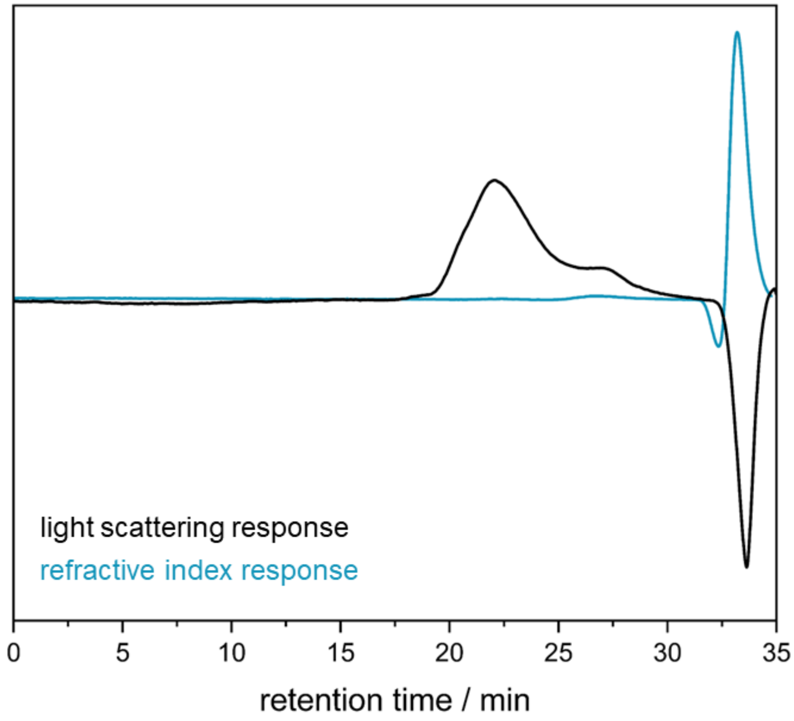

Figure S17: SEC-MALS of polyvinyl phosphonate **P9**.

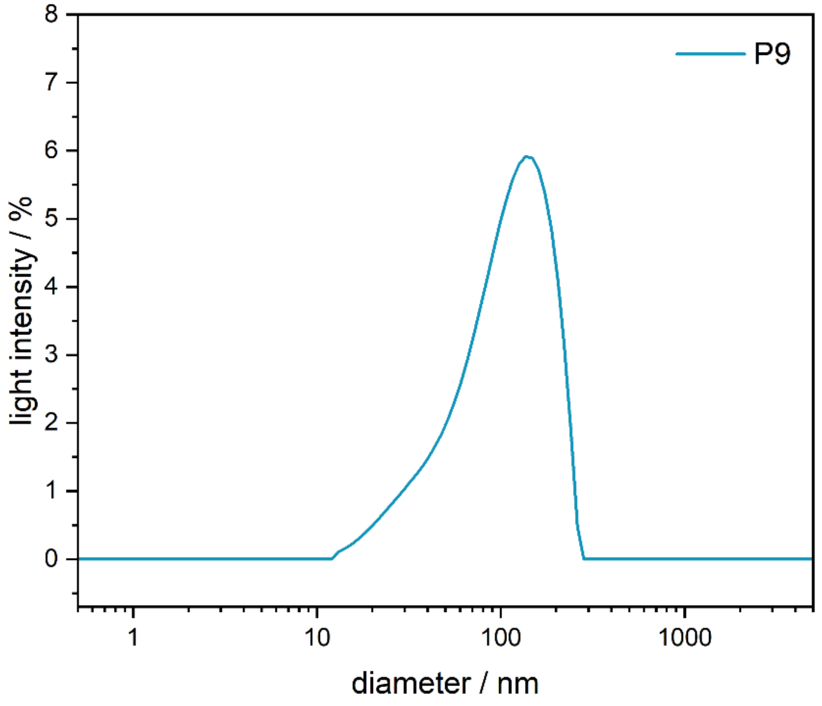

Figure S18: DLS spectra of **P9** (d = 164 ± 21.1 nm, PDI = 0.467).

.

MODIFICATION OF DPRVP VIA ALKYNE-AZIDE CYCLOADDITION (AAC)

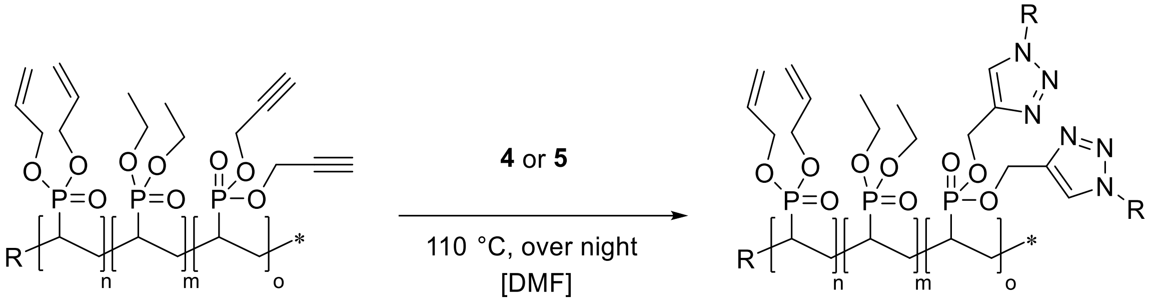

P4

P5 (R= Bn)
P6 (R= hydroxycoumarin)

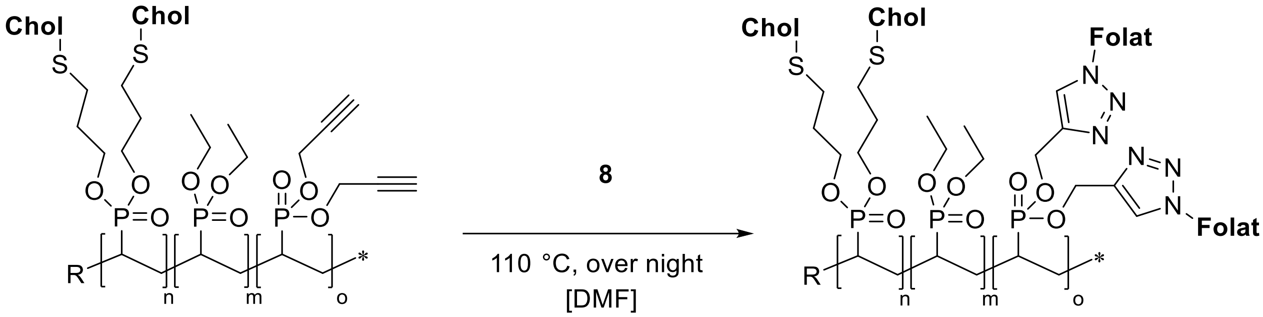

P9 **P10**

The polymer and the respective azide (3.0 eq. per propargyl group) were dissolved in *N*,*N*-dimethylformamid (5.00 mL solvent per 100 mg polymer) before the reaction solution was stirred for 48 hours at 110 °C. The solvent was removed under reduced pressure, and the crude product was purified in different ways.

Modification of P4 with benzylazide 4 = P5

The crude polymer **P5** was dissolved in water and ethanol (water/ethanol = 5/1) and purified by dialysis. In the beginning, against water/ethanol = 5/1, then against water. The functionalized substrate is yielded through freeze-drying.

^{1}H-NMR (400 MHz, MeOD, 500K): δ (ppm) = 8.57 (s, $H_{triazol}$), 7.69 – 7.24 (m, $H_{ar, Benzyl}$), 6.01 (s, -OCH_2CHCH_2), 5.47 – 5.20 (m, -OCH_2CHCH_2), 4.60 (s, -OCH_2CHCH_2), 4.18 (s, -OCH_2CH_3), 2.90 – 1.16 (m, backbone, OCH_2CH_3,-$OCH_2C{\equiv}CH$).

^{31}P-NMR (400 MHz, MeOD, 300K): δ (ppm) = 33.2.

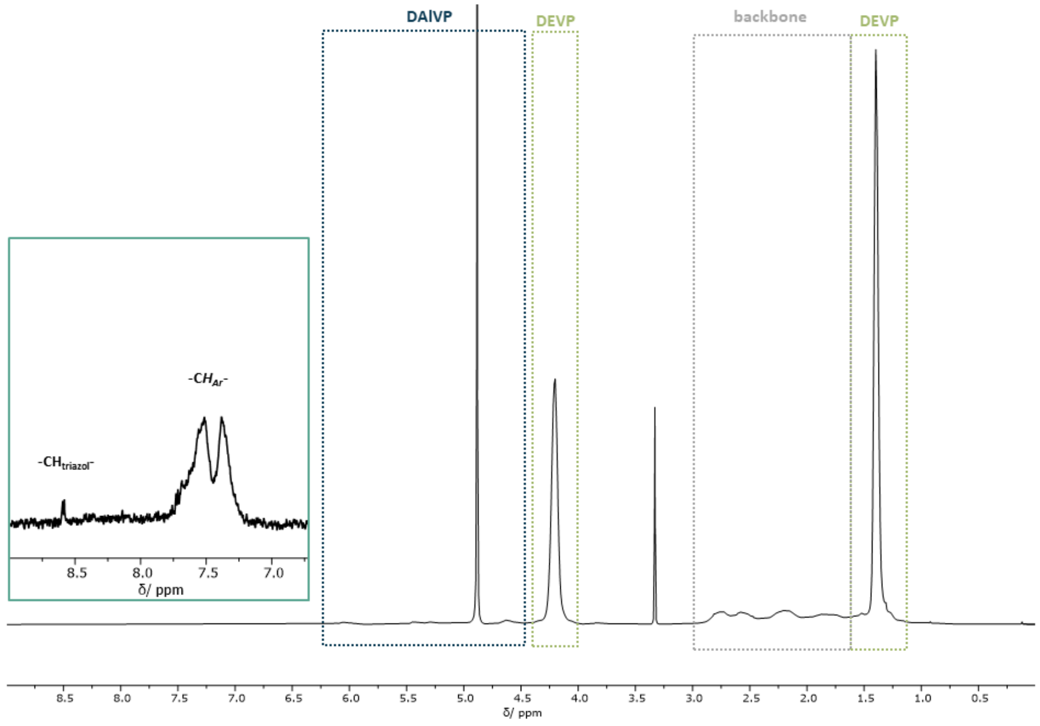

Figure S19: ^{1}H-NMR (400 MHz, MeOD, 300K) of the polyvinyl phosphonates **P5.**

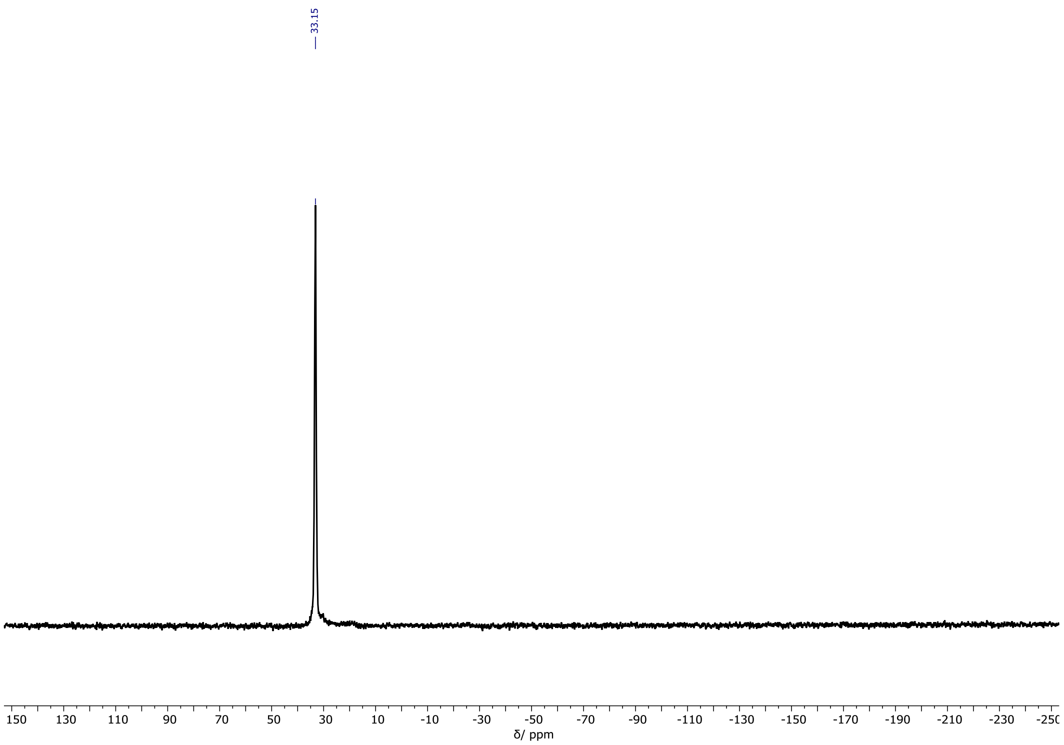

Figure S20: ^{31}P-NMR (203 MHz, $CDCl_3$, 300K) of polyvinyl phosphonate **P5**.

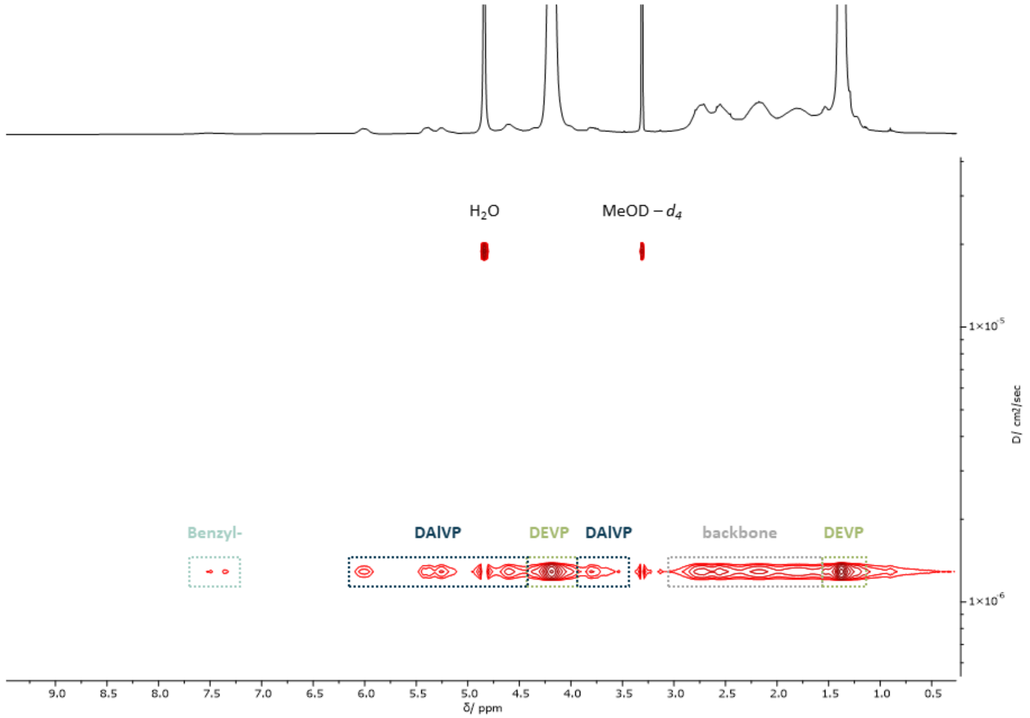

Figure S21: DOSY-NMR of polyvinyl phosphonate **P5.**

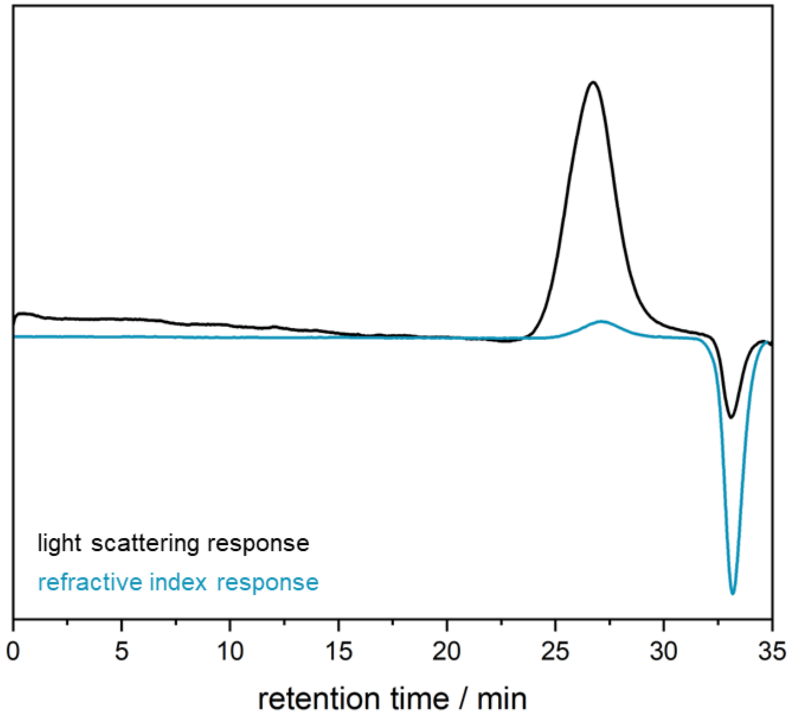

Figure S22: SEC-MALS of polyvinyl phosphonate **P5**.

Modification of P4 with 3-azide-7-hydroxycoumarin 5 = P6

The polymer **P6** was purified via dialysis against water before freeze-drying.

^{1}H-NMR (400 MHz, MeOD, 500K): δ (ppm) = 8.47 (s, $H_{triazol}$), 7.79 – 7.34 (m, $H_{ar,\ Coumarin}$), 7.03 – 6.62 (m, $H_{ar,\ Coumarin}$), 6.00 (s, -OCH_2CHCH_2), 5.51 – 5.15 (m, -OCH_2CHCH_2), 4.58 (s, CH_2CHCH_2), 4.18 (s, -OCH_2CH_3), 2.91 – 1.15 (m, backbone, OCH_2CH_3,-$OCH_2C{\equiv}CH$).

^{31}P-NMR (400 MHz, MeOD, 300K): δ (ppm) = 33.1.

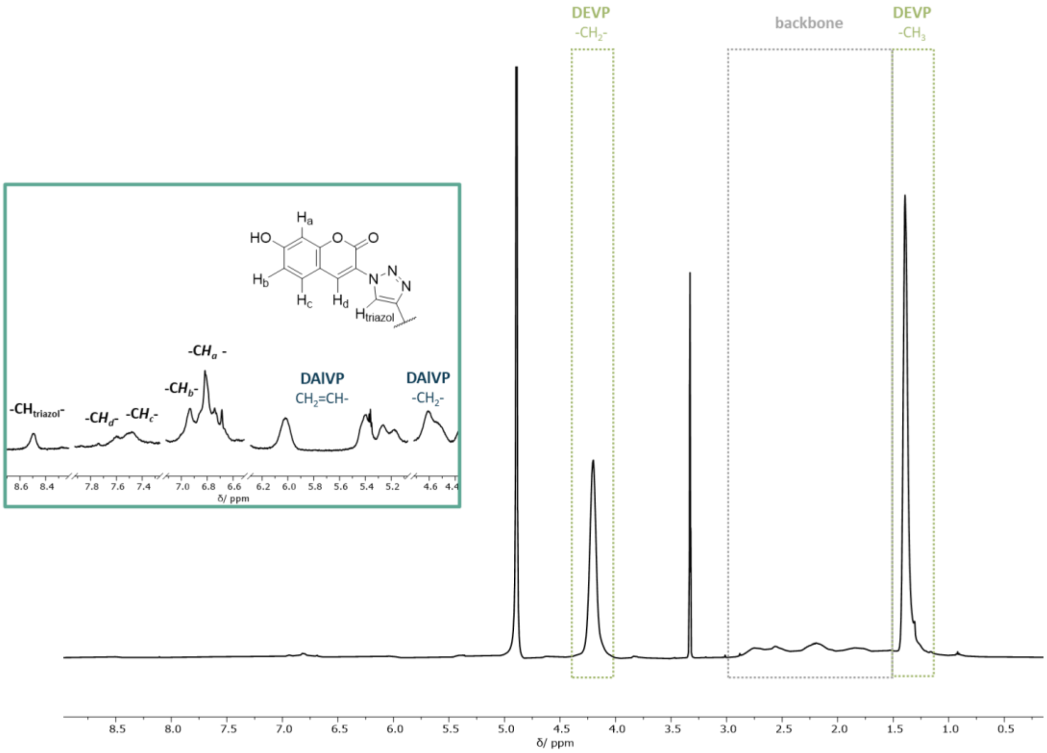

Figure S23: ^{1}H-NMR (400 MHz, $CDCl_3$, 300K) of the polyvinyl phosphonates **P6.**

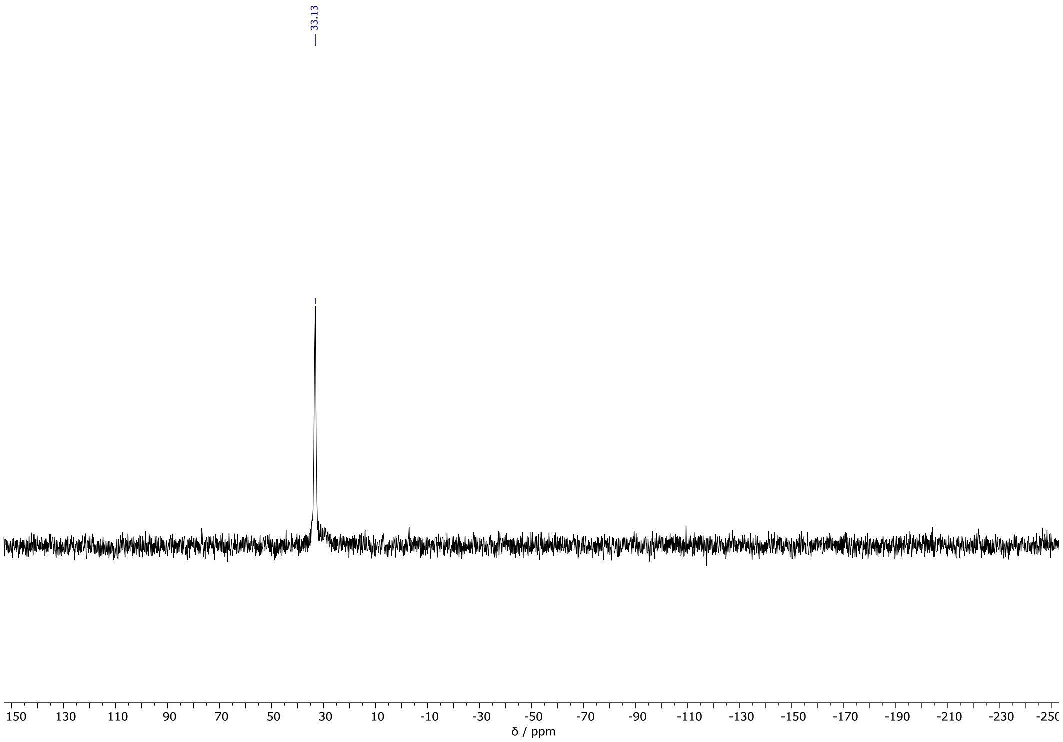

Figure S24: ^{31}P-NMR (203 MHz, MeOD, 300K) of polyvinyl phosphonate **P6.**

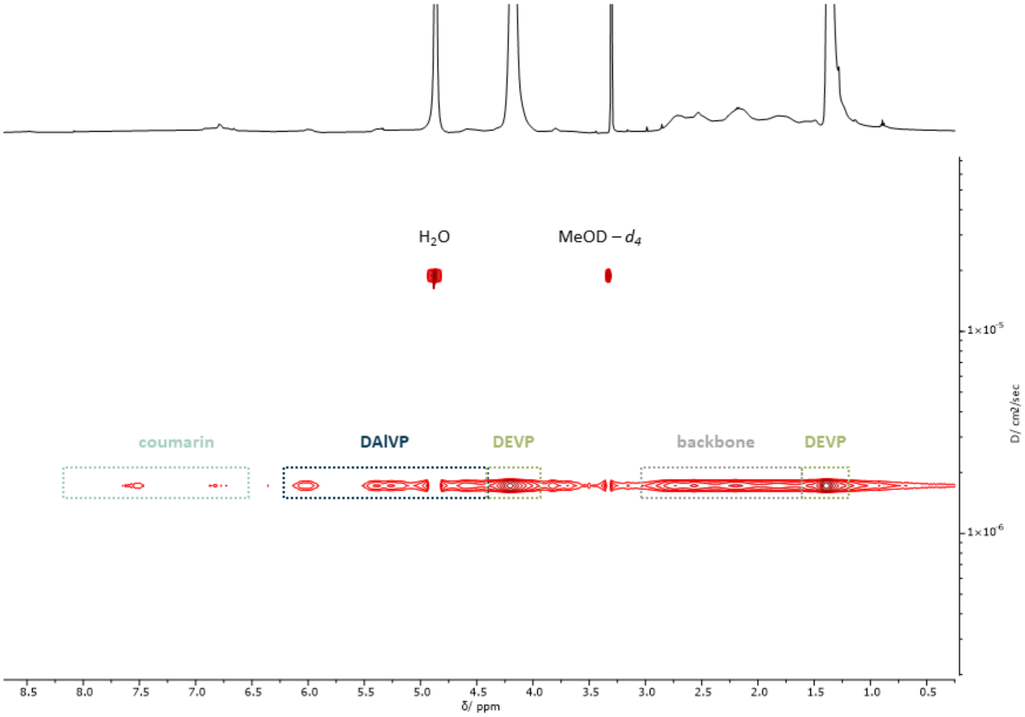

Figure S25: DOSY-NMR of polyvinyl phosphonate **P6.**

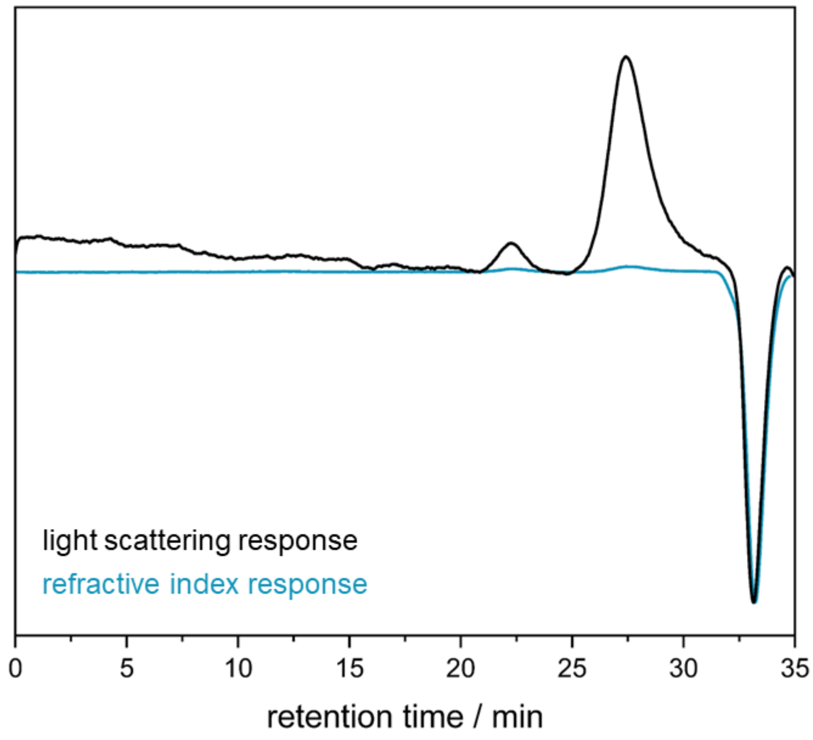

Figure S26: SEC-MALS of polyvinyl phosphonate **P6**.

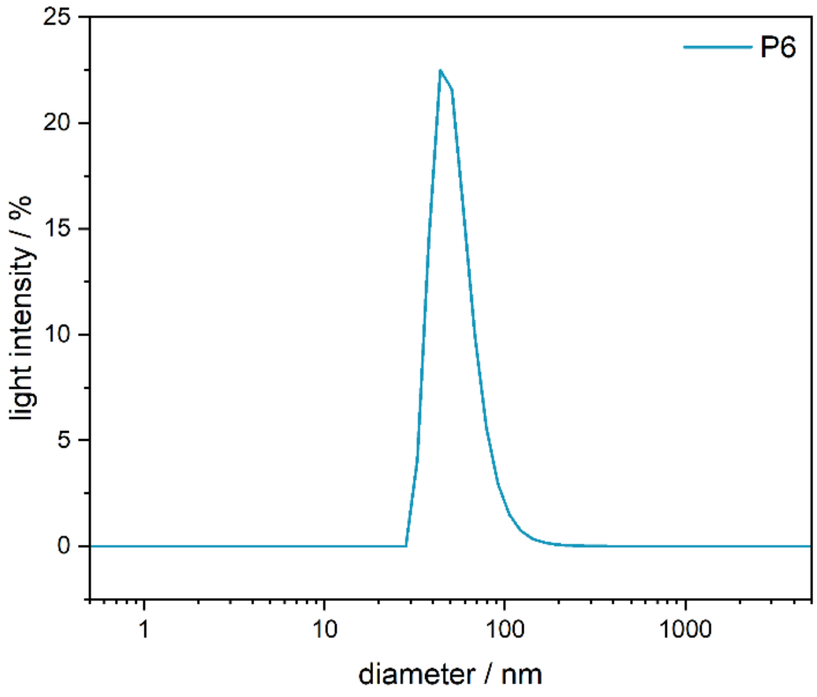

Figure S27: DLS spectra of **P6 (**d = 97.8 ± 0.50 nm, PDI = 0.17).

Modification of P9 with folic acid azide 8 = P10

The crude polymer **P10** was dissolved in a NaOH solution (1 M) and purified by dialysis. In the beginning, against NaOH solution (1 M), then against water. The functionalized substrate is yielded through freeze-drying.

^{1}**H-NMR** (400 MHz, DMSO-*d6*, 500K): *δ* (ppm) = 8.47 – 6.48 (m, H_{Folate}), 7.61 (s, H_{triazol}), 4.01 (s, $-OC\mathit{H}_2CH_3$), 2.78 – 0.99 (m, backbone, $OC\mathit{H}_2CH_{3,}$ $H_{cholesterol}$), 0.99 – 0.71 (m, $-CH3_{3,\ cholesterol}$).

^{31}**P-NMR** (400 MHz, DMSO-*d6*, 300K): *δ* (ppm) = 32.9.

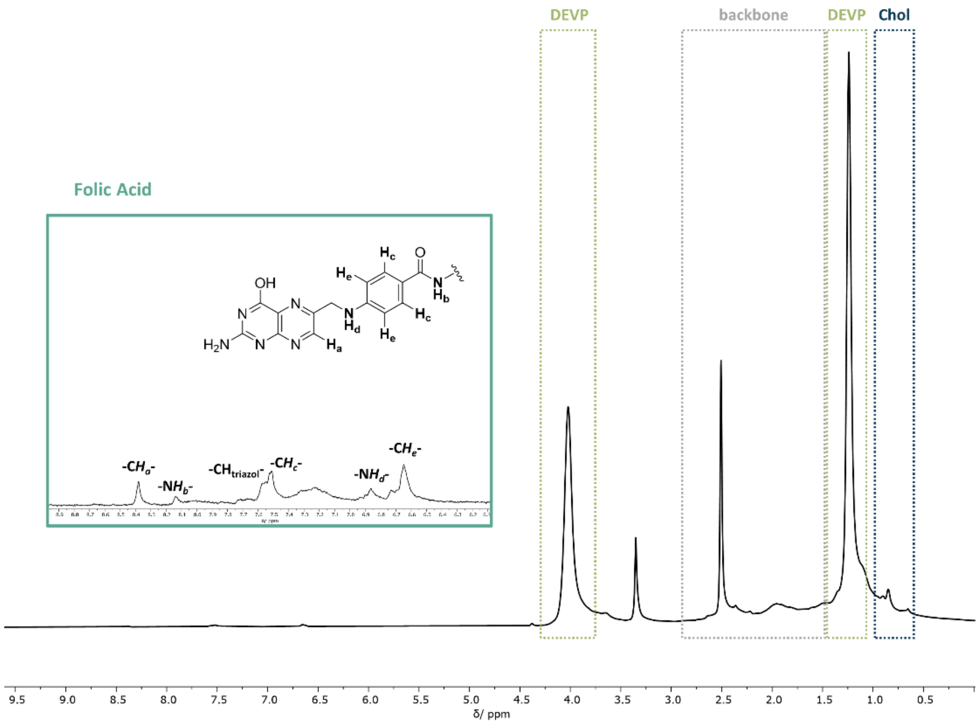

Figure S28: ^{1}H-NMR (400 MHz, DMSO-*d6*, 300K) of the polyvinyl phosphonates **P10.**

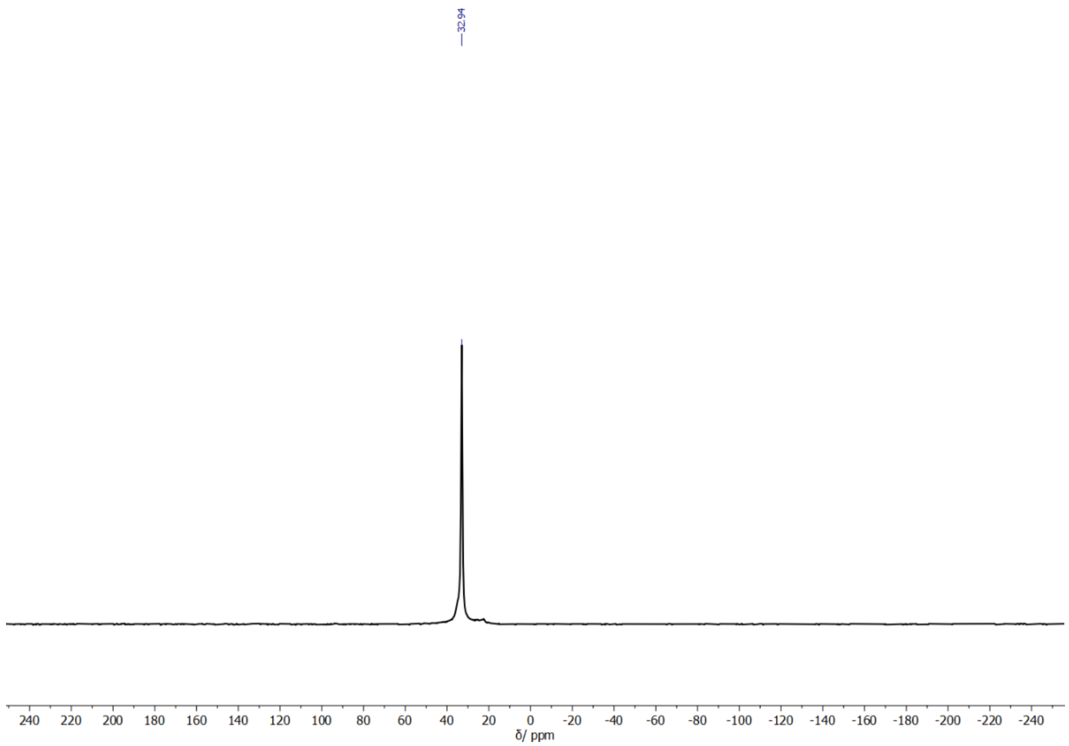

Figure S29: ^{31}P-NMR (203 MHz, DMSO-*d6*, 300K) of polyvinyl phosphonate **P10.**

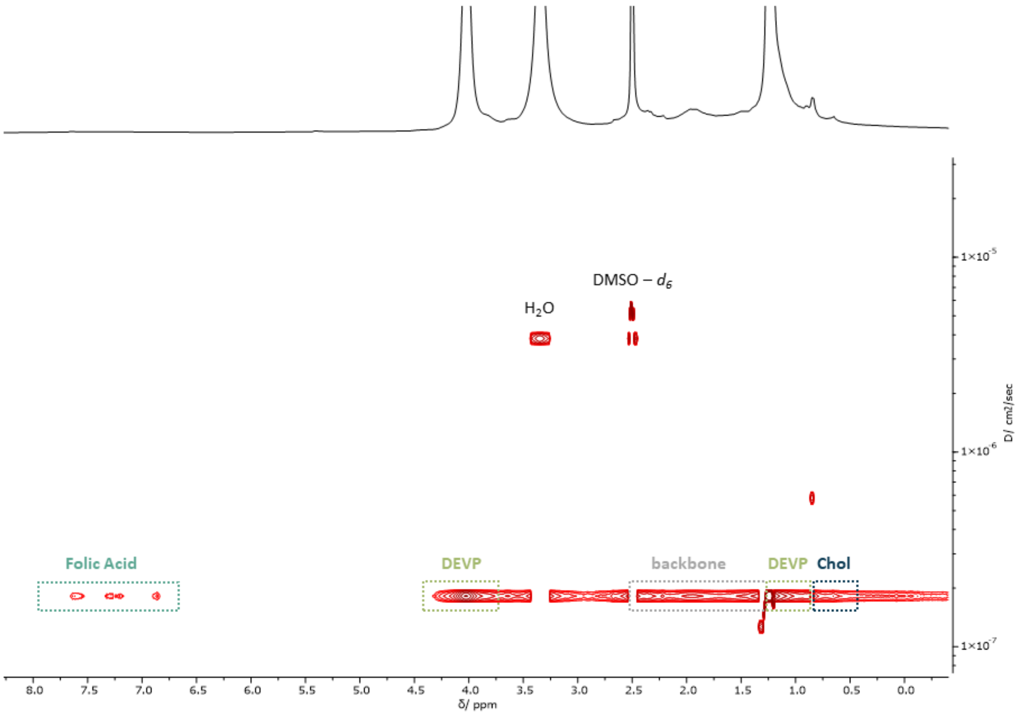

Figure S30: DOSY-NMR of polyvinyl phosphonate **P10.**

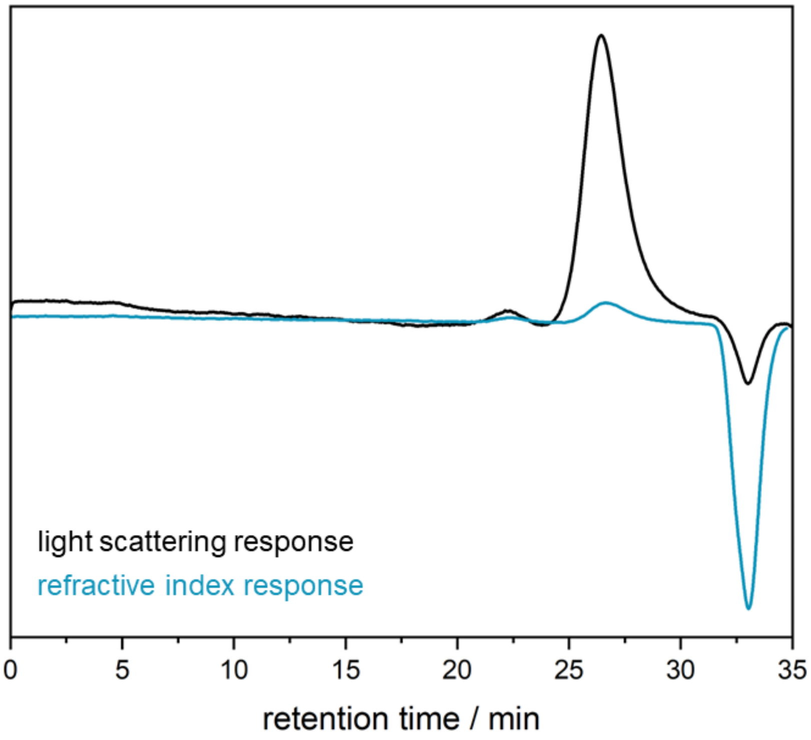

Figure S31: SEC-MALS of polyvinyl phosphonate **P10**.

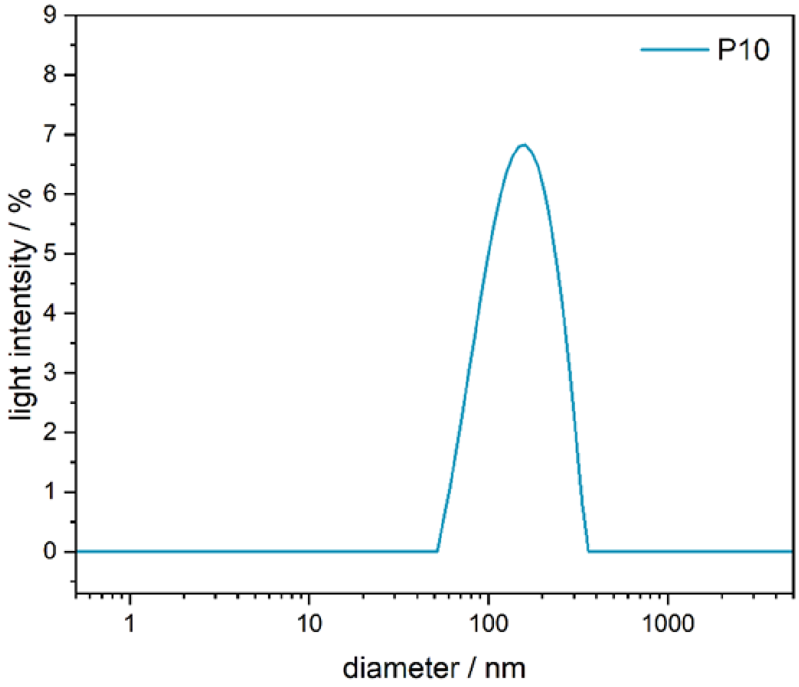

Figure S32: DLS spectra of **P10** (d = 183 ± 8.70 nm, PDI = 0.433).

FUNCTIONALIZATION OF DALVP VIA THIOL-EN CLICK REACTION

MODIFICATION OF P5 WITH CYSTEIN P7

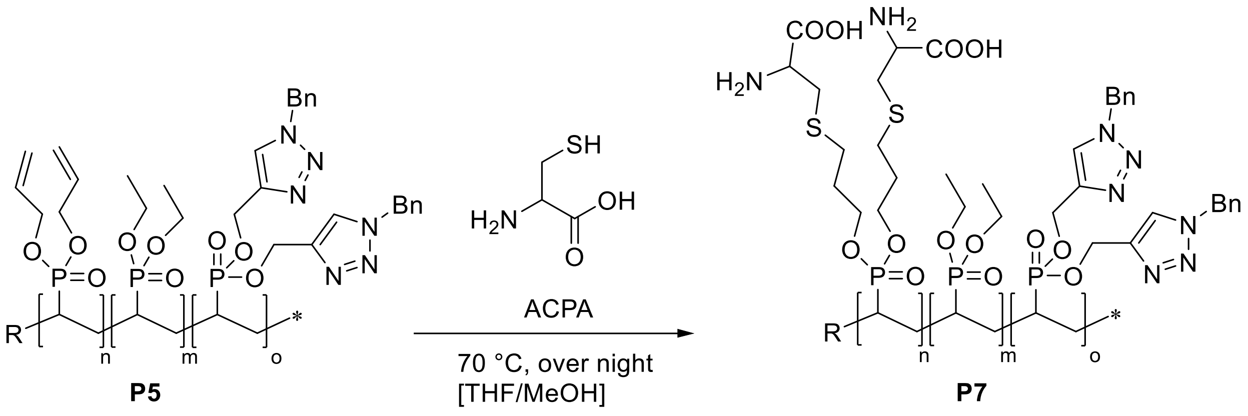

Polyvinyl phosphonate **P5**, L-cysteine (2.0 eq. per allyl group in the polymer), and 4,4'-azobis(4-cyanopentanoic acid) (ACPA) (0.1 eq. per allyl group in the polymer) were dissolved in 25.0 mL water. The mixture was degassed via repeated evacuation and filling with argon (20 iterations) before heating at 70 °C for 15 hours. After purification by dialysis against water, the aqueous solution was freeze-dried to yield the functionalized substrates **P7**.

^{1}H-NMR (400 MHz, MeOD, 300K): δ (ppm) = 8.57 (s, $H_{triazol}$) 7.69 – 7.24 (m, $H_{ar,\ Benzyl}$), 4.18 (s, -OCH_2CH_3), 2.90 – 1.16 (m, backbone, $OC\mathit{H_2}CH_3$,-$OCH_2C{\equiv}C\mathit{H}$).

^{31}P-NMR (400 MHz, MeOD, 300K): δ (ppm) = 33.2.

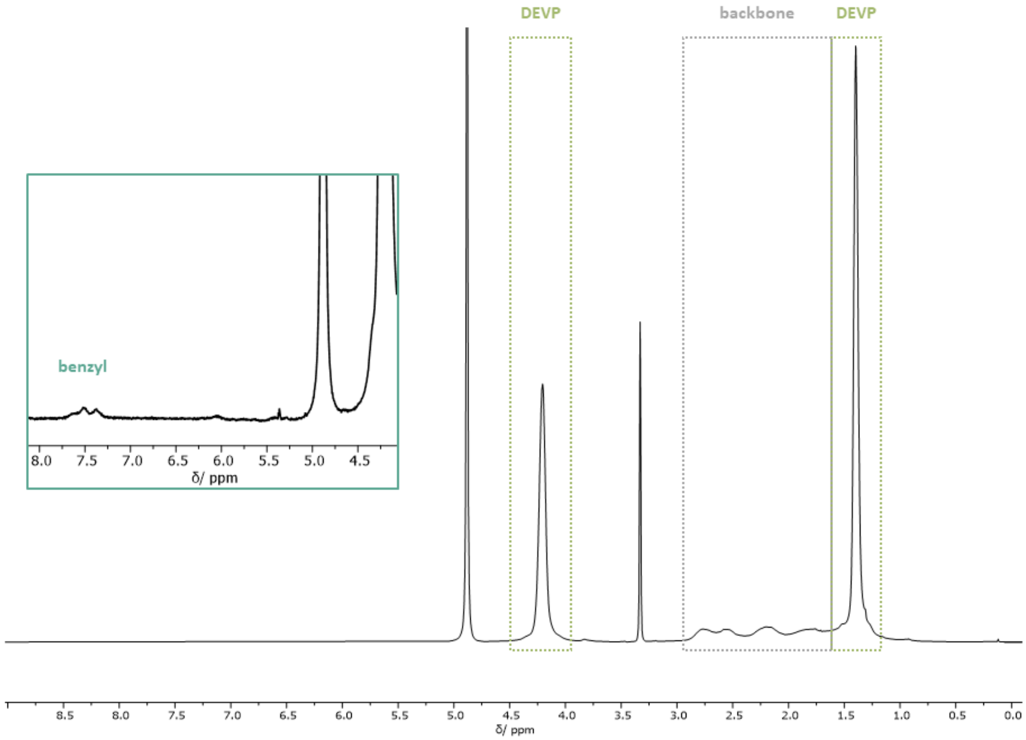

Figure S33: ^{1}H-NMR (400 MHz, MeOD, 300K) of the polyvinyl phosphonates **P7.**

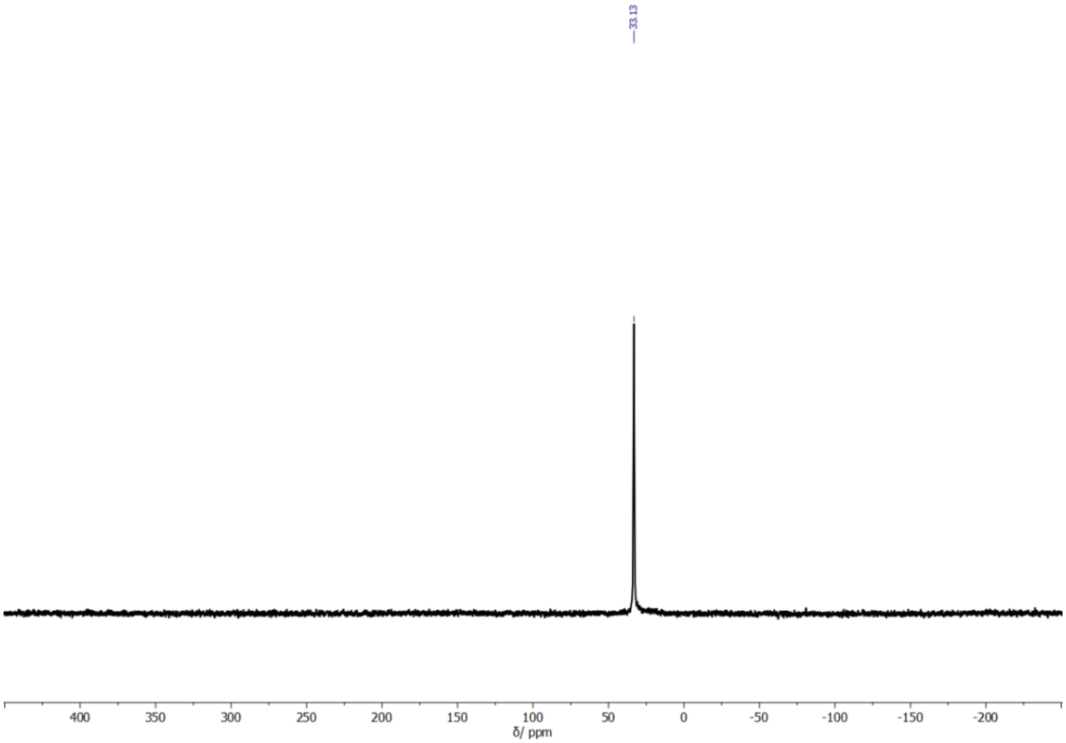

Figure S34: ^{31}P-NMR (203 MHz, MeOD, 300K) of polyvinyl phosphonate **P7.**

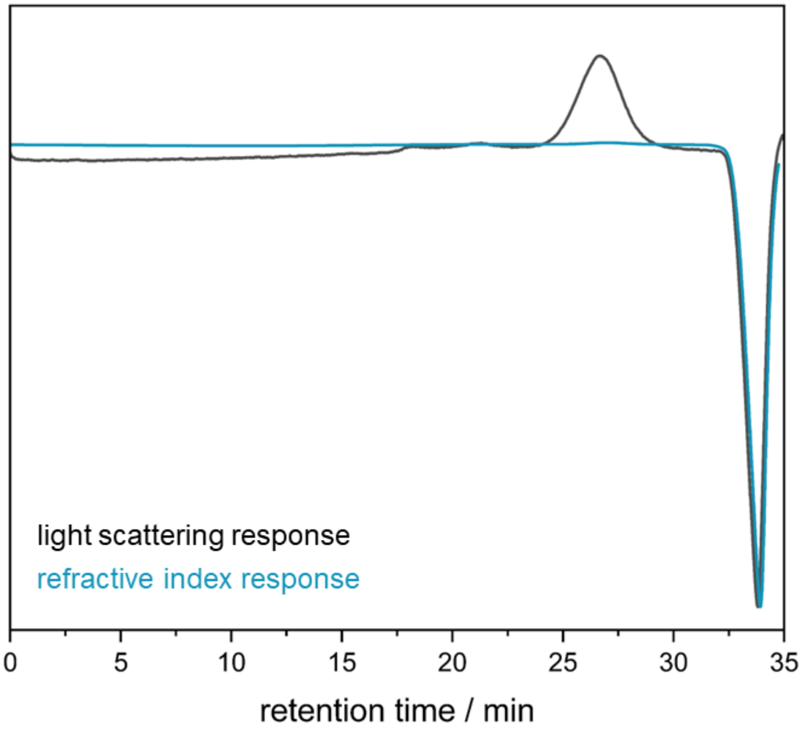

Figure S35: SEC-MALS of polyvinyl phosphonate **P7**.

MODIFICATION OF P3 WITH THIOCHOLESTEROL P8

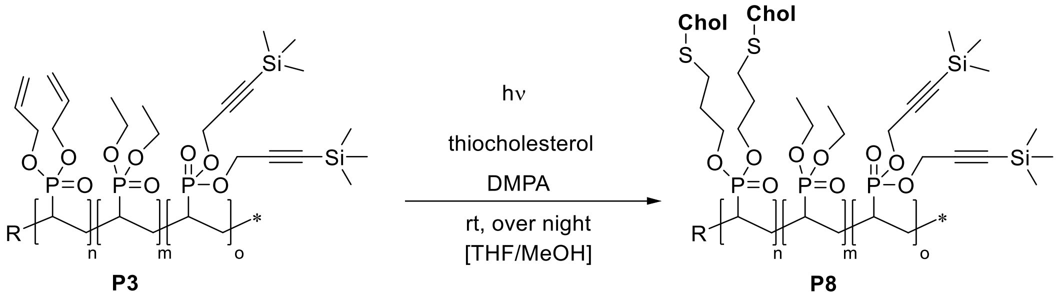

The polymer **P3** was dissolved in a mixture of tetrahydrofuran and methanol (THF/MeOH = 5/1) (15 mL solvent per 100 mg polymer). The thiocholesterol (0.9 eq. per allyl group in the polymer) and 2,2-dimethoxy-2-phenylacetophenone (DMPA) (0.1 eq. per allyl group in the polymer) were added. The mixture was degassed via repeated evacuation and filling with argon (20 iterations) before irradiating (λ = 365 nm) for 18 hours at room temperature. After removing the solvent under reduced pressure, the residue was dissolved in water and tetrahydrofuran (H_2O/THF = 95/5) and purified by dialysis against water. The aqueous solution was freeze-dried to yield the functionalized substrates.

^{1}H-NMR (400 MHz, $CDCl_3$, 500K): *δ* (ppm) = 6.89 – 6.70 (m, $H_{ar,sym\text{-Collidin}}$), 5.92 (s, -$OCH_2C\mathit{H}CH_2$), 5.50 – 5.06 (m, -$OCH_2CHC\mathit{H}_2$), 4.71 (s, -$C\mathit{H}_2C{\equiv}CSi(CH_3)_3$)), 4.54 (s, -$OC\mathit{H}_2CHCH_2$), 4.12 (s, -$OC\mathit{H}_2CH_3$), 2.79 – 0.98 (m, backbone, $OC\mathit{H}_2CH_3$, $H_{cholesterol}$), 0.99 – 0.81 (m, -$CH3_{3,\ cholesterol}$), 0.13 (s, -$CH_2C{\equiv}CSi(C\mathit{H}_3)_3$).

^{31}P-NMR (400 MHz, $CDCl_3$, 300K): *δ* (ppm) = 33.2.

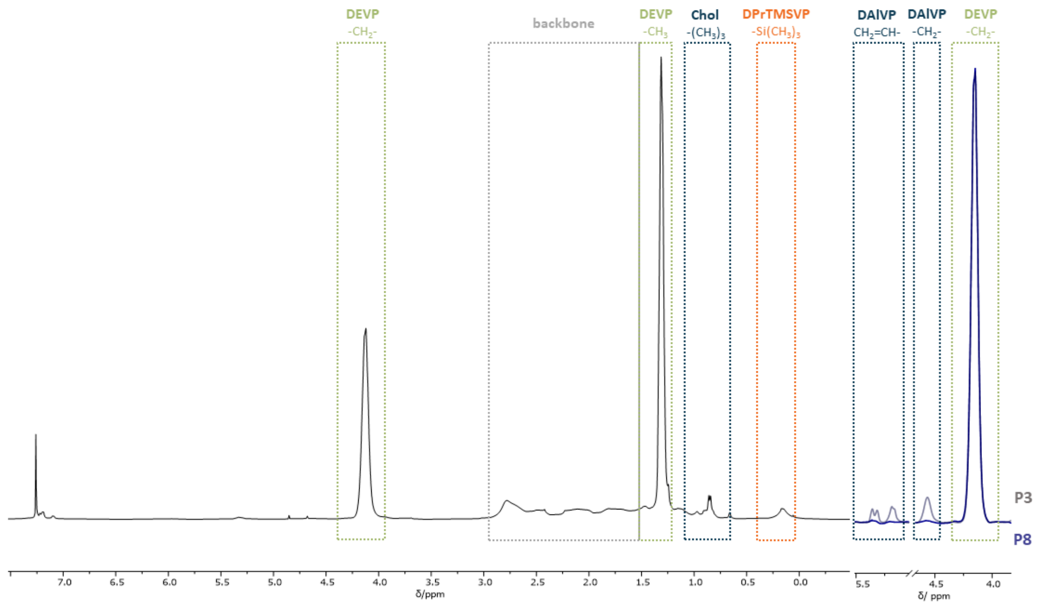

Figure S36: ^{1}H-NMR (400 MHz, $CDCl_3$, 300K) of the polyvinyl phosphonates **P8.**

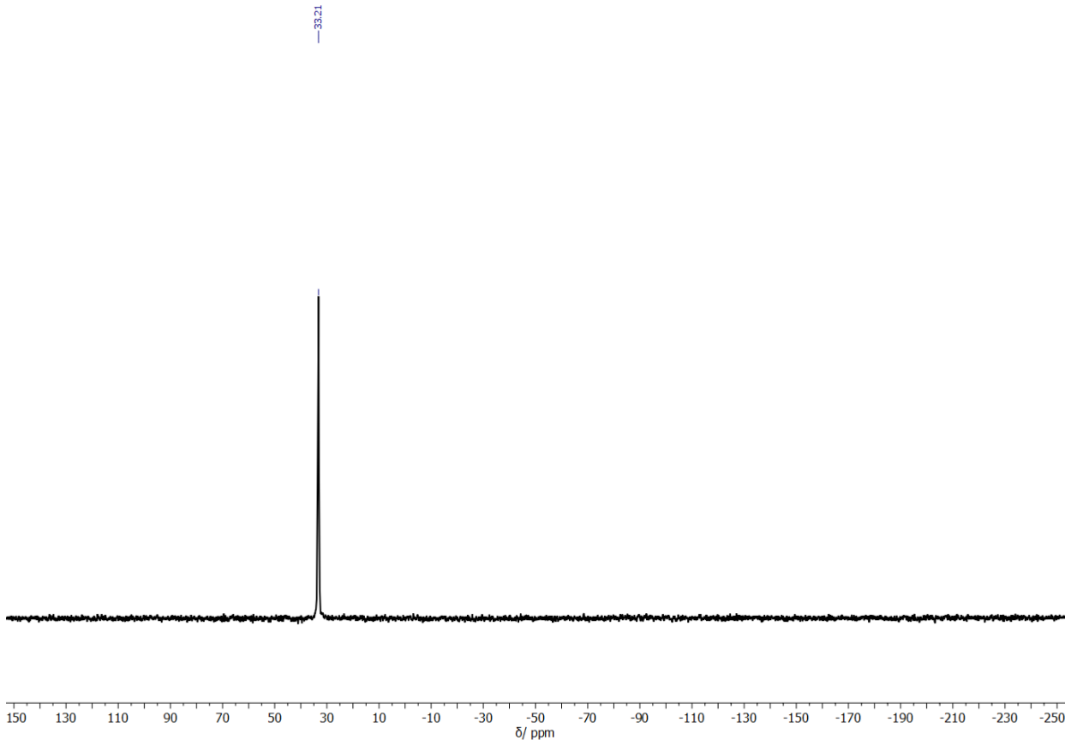

Figure S37: ^{31}P-NMR (203 MHz, $CDCl_3$, 300K) of polyvinyl phosphonate **P8.**

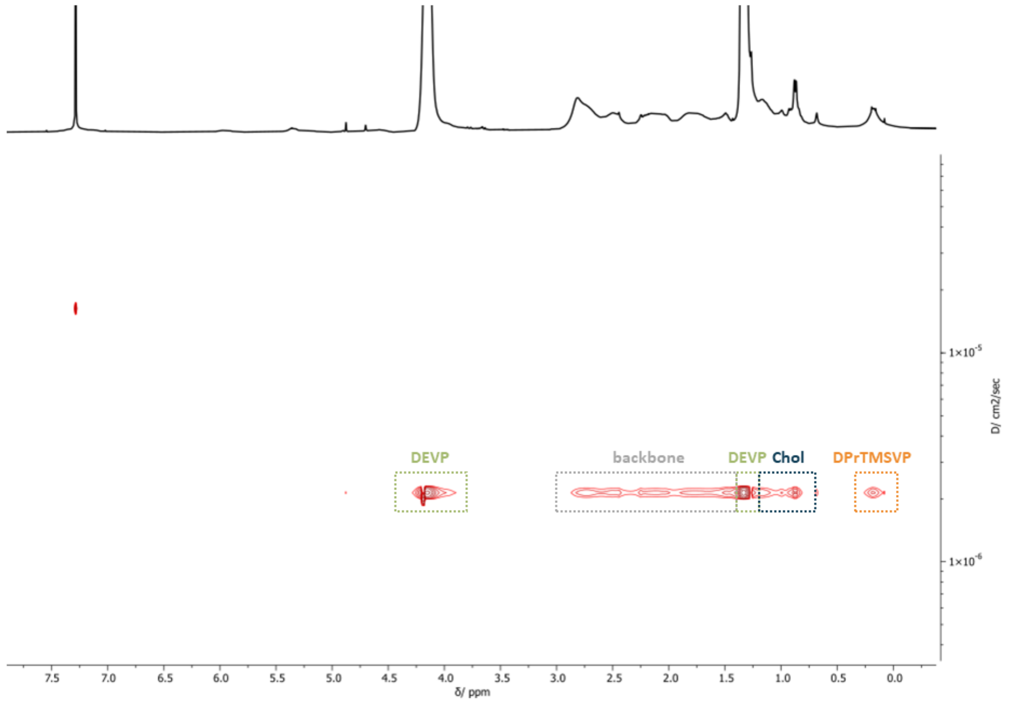

Figure S38: DOSY-NMR of polyvinyl phosphonate **P8.**

5. REFERENZES

1. K. C. Hultzsch, P. Voth, K. Beckerle, T. P. Spaniol and J. Okuda, *Organometallics*, 2000, **19**, 228-243.
2. S. Salzinger, B. S. Soller, A. Plikhta, U. B. Seemann, E. Herdtweck and B. Rieger, *Journal of the American Chemical Society*, 2013, **135**, 13030-13040.
3. C. Schwarzenböck, P. J. Nelson, R. Huss and B. Rieger, *Nanoscale*, 2018, **10**, 16062-16068.
4. T. M. Pehl, F. Adams, M. Kränzlein and B. Rieger, *Macromolecules*, 2021, **54**, 4089-4100.
5. Q. Yang, C. Draghici, J. T. Njardarson, F. Li, B. R. Smith and P. Das, *Organic & Biomolecular Chemistry*, 2014, **12**, 330-344.
6. C. Schwarzenböck, A. Schaffer, P. Pahl, P. J. Nelson, R. Huss and B. Rieger, *Polymer Chemistry*, 2018, **9**, 284-290.
7. L. Liu, M. Zheng, T. Renette and T. Kissel, *Bioconjugate Chemistry*, 2012, **23**, 1211-1220.
8. G. A. Limb, T. E. Salt, P. M. Munro, S. E. Moss and P. T. Khaw, *Investigative ophthalmology & visual science*, 2002, **43**, 864-869.
9. T. M. Pehl, M. Kränzlein, F. Adams, A. Schaffer and B. Rieger, *Catalysts*, 2020, **10**, 448.
